# Piezo1-driven mechanotransduction regulates mitochondrial biogenesis by AMPK/SIRT1-mediated PGC-1α deacetylation to ameliorate bone loss in disuse osteoporosis

**DOI:** 10.7150/ijbs.124043

**Published:** 2026-01-01

**Authors:** Jianpeng Chen, Dengying Wu, Chengbin Huang, Zijian Yan, Jiahao Wang, Siteng Li, Xuankuai Chen, Yanbin Zhu, Yingze Zhang

**Affiliations:** 1 Nankai University, Tianjin, China.; 2 Department of Orthopaedics, The Third Hospital of Hebei Medical University, Shijiazhuang, Hebei Province, China.; 3 Department of Orthopaedics, The Second Affiliated Hospital and Yuying Children's Hospital of Wenzhou Medical University, Wenzhou, Zhejiang Province, China.

**Keywords:** Piezo1, disuse osteoporosis, AMPK/SIRT1, PGC-1α deacetylation, mitochondrial biogenesis

## Abstract

Disuse osteoporosis (DOP), a skeletal disorder triggered by insufficient mechanical loading, manifests as progressive bone mass deterioration and microarchitectural weakening. Piezo1, a key mechanosensitive ion channel expressed in bone cells, is implicated in maintaining skeletal homeostasis. Using a murine hindlimb unloading (HLU) model simulating microgravity-induced bone loss, we observed significant downregulation of Piezo1 expression in bone tissue and isolated bone marrow-derived mesenchymal stem cells (BMSCs). Systemic administration of the Piezo1 agonist Yoda1 attenuated HLU-induced osteopenia and improved bone formation capacity. Mechanistic studies in BMSCs demonstrated that Piezo1 activation promoted mitochondrial biogenesis. This effect required AMPK/SIRT1 signaling-dependent deacetylation of PGC-1α, leading to enhanced mitochondrial function, improved osteogenic differentiation, and reduced apoptosis. Critically, pharmacologic inhibition of SIRT1 abolished the osteoprotective effects of Yoda1 in vivo. These findings establish that mechanical unloading impairs Piezo1-mediated mechanotransduction in BMSCs, contributing to disrupted skeletal homeostasis, which can be mitigated by exogenous Piezo1 activation. Our results define a mechanism where Piezo1 integrates mechanical signals into the AMPK/SIRT1/PGC-1α signaling cascade to regulate mechanoadaptive bone formation, highlighting Piezo1 activation as a potential mechanism-based therapeutic strategy for disuse osteoporosis.

## Introduction

Disuse osteoporosis (DOP) is a prevalent clinical disorder characterized by progressive bone loss and microarchitectural deterioration, leading to increased fracture risk. It arises primarily from diminished mechanical stimulation of bone tissue, commonly associated with prolonged immobilization, bed rest, microgravity exposure, or neuromuscular impairments due to injury or neurological conditions^1^, ^2^. Under physiological conditions, mechanical loading exerts critical anabolic effects on the skeleton. It promotes the osteogenic differentiation of bone marrow mesenchymal stem cells (BMSCs) and inhibits osteocyte-mediated osteoclastogenesis, thereby maintaining a positive bone balance. Conversely, the absence of mechanical stimulation disrupts this equilibrium^3^, ^4^. The precise molecular mechanisms linking mechanical unloading to dysregulated bone remodeling remain incompletely understood, and effective pharmacotherapies are currently lacking. Elucidating the mechanotransduction pathways governing skeletal homeostasis is therefore critical for developing novel therapeutic strategies against DOP.

As an important cell type regulating bone mass in the bone microenvironment, BMSCs and their osteogenic progeny exhibit high sensitivity to mechanical stimuli, including stress, strain, cyclic stretching, and fluid shear stress (FSS)^5^-^7^. These mechanical cues play a fundamental role in regulating BMSCs fate decisions, particularly osteogenic differentiation. The mechanosensitive ion channel Piezo1, expressed in various bone cell types including BMSCs, serves as a critical transducer of physical forces into biochemical signals. It has been implicated in sensing stimuli like hydrostatic pressure (HP) and regulating load-induced bone remodeling^8^, ^9^. Nevertheless, how BMSCs convert mechanical stimuli into biochemical signals that result in abnormal bone remodeling is poorly understood.

Recent evidence increasingly highlights the critical role of mitochondrial dynamics and function in mesenchymal stem cell (MSC) biology^10^. The absence of mechanical stimulation in microgravity impairs osteoblast function and compromises mitochondrial metabolic function, thereby disrupting cellular energy homeostasis^11^. Piezo1 regulates osteoblast differentiation and ATP energy metabolism through the AMPK-ULK1 axis^12^. Peroxisome proliferator-activated receptor γ coactivator 1-α (PGC-1α) serves as a central regulator of cellular energy metabolism, playing a pivotal role in mitochondrial biogenesis and the cellular defense against oxidative stress^13^. It is a key regulator of the bone formation process^14^, ^15^. Full activation of PGC-1α is mediated by its phosphorylation by AMPK (adenosine 5'-monophosphate-activated protein kinase) and deacetylation by SIRT1 (silencing information regulator 1)^16^. Mitochondrial biogenesis is the cellular process responsible for increasing mitochondrial mass and function, essential for meeting energy demands and maintaining homeostasis. The transcriptional coactivator PGC-1α serves as the master regulator^17^. The stiffness of the extracellular matrix modulates the osteogenic differentiation of mesenchymal stem cells by regulating mitochondrial biogenesis^18^. However, the role of Piezo1 in BMSCs and its regulation mechanism to mitochondria have not been fully elucidated.

Based on the established role of Piezo1 in mechanotransduction and the critical function of mitochondria in osteogenesis, we hypothesized that mechanical unloading induces bone loss, at least in part, by downregulating Piezo1 expression in BMSCs, thereby disrupting mitochondrial biogenesis via the AMPK/SIRT1/PGC-1α signaling axis. We used hindlimb unloading mice to create the disuse osteoporosis model. Our study was designed to (1) determine whether mechanical unloading alters Piezo1 expression and impairs mitochondrial function in BMSCs, (2) investigate if pharmacological activation of Piezo1 by Yoda1 can rescue osteogenic differentiation and mitochondrial biogenesis in mechanically unloaded BMSCs, (3) elucidate whether the AMPK/SIRT1/PGC-1α signaling axis is both necessary and sufficient for mediating the effects of Piezo1 activation, (4) establish whether SIRT1-dependent signaling mediates the protective effects of Yoda1 in a murine model of disuse osteoporosis.

## Materials and Methods

### Animals

Three-month-old male C57BL/6 mice (25-30 g) (SPF Biotechnology Co., Ltd., Beijing, China) were acclimatized and maintained under specific pathogen-free (SPF) conditions at the Animal Experiment Center of Hebei Medical University (Shijiazhuang, Hebei, China). To establish a simulated microgravity environment, the hindlimb unloading (HLU) model was implemented following standard protocols. Briefly, mice were individually housed in cages and suspended at a 30° head-down tilt position using a harness system. The experimental configuration allowed libitum access to standard rodent chow and water throughout the study period. Animal welfare was assessed through daily monitoring of body weight, food consumption, and tail integrity, with comprehensive health evaluations conducted every 48 hours. All experiments in this study were approved by the Ethics and Welfare Committee for Laboratory Animals of Hebei Medical University (IACUC number: Hebmu-2025045).

### BMSCs isolation, culture, and identification

After 2 weeks of limb unloading, the BMSCs were isolated from the unloading femurs and tibias. Meanwhile, BMSCs were isolated from age-matched mice subjected to mechanical loading using the same method. Mice were euthanized via cervical dislocation and surface-sterilized with 75% ethanol. Under strict aseptic conditions, the femurs and tibias were dissected. Bone marrow was flushed into centrifuge tubes using Minimum Essential Medium α (MEM-α) with 10% fetal bovine serum (FBS) and 1% penicillin/streptomycin. The resulting cell suspension was filtered through a 0.7-μm membrane filter and centrifuged at 400g for 5 minutes at room temperature (RT). After discarding the supernatant, 1 ml of red blood cell lysis buffer was added to the pellet. Cells were resuspended thoroughly and incubated for 5 minutes at RT. The suspension was centrifuged again at 400g for 5 minutes (RT), and the supernatant discarded. The isolated BMSCs were then seeded in the above culture medium for subsequent culture and experiments, all experiments used passage 3 (P3) BMSCs. BMSCs identity was confirmed by flow cytometry analysis of surface markers.

For immunophenotyping, BMSCs were incubated in the dark for 30 minutes with APC-conjugated antibodies against CD34 (E-AB-F1284E, Elabscience, China), CD45 (103111, Biolegend, USA), CD73 (127209, Biolegend, USA), CD90 (104311, Biolegend, USA), and CD105 (120413, Biolegend, USA), each at a 1:100 dilution. Cells were then washed, collected, and analyzed using a flow cytometer (BD Accuri^TM^ C6 Plus, USA). Data analysis was performed using FlowJo software (V10.7.1) (Figure S1).

### Drug administration

Mice were divided into a Sham group (normal loading) and an HLU group (hindlimb unloading). To evaluate the therapeutic potential of Yoda1 (HY-18723, MedChemExpress, USA) in unloading-induced bone loss, HLU mice were further randomized into three groups based on previous studies^19^, ^20^: HLU, HLU + Yoda1 (2.5 mmol/kg), and HLU + Yoda1 (5 mmol/kg). Yoda1 was administered once daily by intraperitoneal injection, five days per week until the sacrifice. To validate the underlying mechanism in vivo, an additional group (HLU + Yoda1 + EX-527) was included, receiving EX-527 (5 mg/kg)^21^ intraperitoneally one hour before Yoda1 injection. The remaining groups received an equal volume of saline.

Bone marrow mesenchymal stem cells (BMSCs) isolated from Sham and HLU mice were initially categorized into their respective Sham and HLU groups. To investigate the therapeutic effect of Piezo1 activation, HLU group cells were further randomized into two treatment subgroups^22^, ^23^: HLU + Yoda1 (2.5 μM) and HLU + Yoda1 (5 μM). Yoda1 was prepared as a 10 mM stock solution in dimethyl sulfoxide (DMSO) and diluted in culture medium immediately before use. The final DMSO concentration did not exceed 0.1%. For mechanistic inhibition studies, cells from the HLU group were pre-treated for 1 hour with specific inhibitors prior to the addition of Yoda1. The inhibitors included: KN-93 (10 μM, a CaMKII inhibitor; HY-15465, MedChemExpress, USA)^24^, GsMTx4 (10 μM, a Piezo1 inhibitor; HY-P1410, MedChemExpress, USA)^25^, EX-527 (10 μM, a SIRT1 inhibitor; HY-15452, MedChemExpress, USA)^26^, and SR-18292 (10 μM, a PGC-1α deacetylation inhibitor; HY-101491, MedChemExpress, USA)^27^. All inhibitors were also dissolved in DMSO and diluted to the final working concentration in culture medium.

### Cell differentiation assay

To induce osteogenic differentiation, BMSCs were seeded in 24-well plates. Upon reaching 70% confluency, the growth medium was replaced with osteogenic induction medium (MUXMX-90021, Cyagen, China). For alkaline phosphatase (ALP) staining, cells were stained according to the manual of BCIP/NBT Alkaline Phosphatase Color Development Kit (C3206, Beyotime, China) on day 7. Mineralized matrix deposition was subsequently evaluated by Alizarin Red S staining (G1452, Solarbio, China) on day 21.

### μCT analyses

The femoral specimen was isolated with subsequent removal of surrounding soft tissues. Micro-computed tomography (micro-CT) scanning was performed using a Bruker Skyscan 1176 system (Bruker, Kontich, Belgium) under the following parameters: 100 kV tube voltage, 98 μA current, and 9 μm isotropic voxel resolution. The images were reconstructed using NRecon (Version 1.6, SkyScan). Subsequent quantitative analysis was performed with CTAn (Version 1.9, SkyScan) to obtain the bone volume fraction (BV/TV, %), trabecular thickness (Tb.Th, μm), trabecular number (Tb.N, mm⁻¹), and trabecular separation (Tb.Sp, μm). Three-dimensional visualization of the femoral architecture was generated using CTVox software (v2.0, SkyScan).

### Hematoxylin & eosin and tartrate resistant acid phosphatase staining

Femoral specimens for histological analysis were processed through standardized tissue preparation protocols. Freshly dissected femurs were immediately fixed in 4% paraformaldehyde (PFA) for 48 hr at 4°C. Following fixation, samples were subjected to decalcification using 0.5 M EDTA (G1105, Servicebio, China) under continuous shaken for 21 days at 25°C. Subsequent tissue processing included sequential dehydration through graded ethanol series (70%-100%), xylene clearing, and paraffin embedding. Femoral sections (4μm thick) were prepared and stained with hematoxylin & eosin (HE) (G1076, Servicebio, China). Additionally, osteoclasts in decalcified bone sections were identified using tartrate resistant acid phosphatase (TRAP) staining according to the manufacturer's protocol (G1050, Servicebio, China).

### Immunohistochemistry

Immunohistochemistry (IHC) of femoral tissues was performed as previously described. Briefly, endogenous peroxidase activity in the sections was quenched using 2.5% hydrogen peroxide. Antigen retrieval was then performed with EDTA Antigen Retrieval solution (C1034, Solarbio, China)at 55°C. After blocking with goat serum, sections were incubated overnight at 4°C with the primary antibody against Piezo1 (15939-1-AP, Proteintech, USA). This was followed by incubation with an horseradish peroxidase (HRP)-conjugated goat anti-rabbit/mouse IgG secondary antibody (G1303, Servicebio, China) at room temperature for 1 hour. Immune complexes were visualized using a DAB substrate kit (ZLI-9018, ZSGB-BIO, China). Images were captured using a light microscope and analyzed using ImageJ software.

### Immunofluorescence

Bone marrow mesenchymal stem cells (BMSCs) were seeded onto cell slides and cultured for 24 hours. Following the respective interventions, cells were fixed with 4% paraformaldehyde, permeabilized with 0.1% Triton X-100, and blocked with 5% goat serum. Subsequently, cells were incubated overnight at 4°C with the following primary antibodies: anti-PGC-1α, anti-SIRT1, anti-TFAM, anti-Runx2, anti-Acetylation, anti-NRF1, anti-TOM20, and anti-dsDNA. Primary antibody dilution ratios are detailed in **Table S1.** The corresponding secondary antibody was incubated at room temperature for 1 hour the next day. Finally, nuclei were counterstained with DAPI at room temperature for 60 seconds. Antigen retrieval for the de-waxed histological sections followed the same protocol as for immunohistochemistry, with subsequent steps matching those of cellular immunofluorescence staining.

### Measurement of cellular ATP levels

Cellular ATP quantification was performed using a commercial ATP Assay Kit (S0026, Beyotime, China) according to the manufacturer's protocol. Luminescence measurements were conducted on a PerkinElmer VICTOR Nivo multimode plate reader (PerkinElmer, Waltham, MA, USA) with final ATP levels normalized to control group values.

### Calcium imaging

Following two washes with ice-cold Hanks Balanced Salt Solution (HBSS; Gibco, USA), each group cells underwent fluorescent labeling by incubation with 1 μM Fluo-4 AM (S1060, Beyotime, China) in HBSS for 30 minutes at 37°C under 5% CO₂, protected from light. Unincorporated dye was removed via two additional HBSS washes (300 × g, 5 min). Calcium imaging was obtained by fluorescence microscopy. Baseline mean fluorescence intensity (MFI) was quantified using a BD Accuri^TM^ C6 Plus flow cytometer (BD Biosciences, USA) equipped with a 488 nm excitation laser. Data analysis was performed using FlowJo 10.6.2 software (BD Biosciences, USA), with fluorescence shifts normalized to baseline values.

### Mitochondria morphology

The cells were seeded onto confocal imaging dishes and stained with MitoTracker Green (C1048, Beyotime, China) at 37 °C for 30 minutes. Mitochondrial morphology was further analyzed using the Mitochondrial Analyzer plugin and Fiji software. The shape factor (FF = perimeter² / (4π × area)) and aspect ratio (AR) are parameters for assessing mitochondrial fragmentation. FF and AR values approaching 1 indicate a more circular mitochondrial shape. Conversely, the morphology becomes more elongated with increasing values.

### Western blot

Total protein was extracted from BMSCs using RIPA lysis buffer, and the protein concentration was determined using a BCA protein assay kit (P0010, Beyotime, China). The proteins were then separated by SDS-PAGE and transferred to a PVDF membrane (IPVH00010, Merck Millipore, Germany). After blocking with 5% skim milk for 2 hours, the membrane was incubated overnight at 4 °C with the appropriate primary antibody, followed by incubation with the corresponding HRP-conjugated secondary antibody (goat anti-mouse IgG (H+L) or goat anti-rabbit IgG (H+L)) at room temperature for 2 hours. Band signals were detected and quantified using Image Lab 3.0 software (Bio-Rad) with Ultrasensitive ECL Detection Kit (G2020, Servicebio, China). Primary antibody dilution ratios are detailed in **Table S1**.

### DNA isolation and mtDNA copy number assay

Relative mitochondrial DNA (mtDNA) levels were quantified by qPCR as previously described^28^, ^29^. Briefly, total DNA was extracted from BMSCs using the MolPure® Cell/Tissue DNA Kit (18700ES50, Yeasen, China). Using 10 ng of DNA per reaction, qPCR was performed with mitochondrial-encoded cytochrome c oxidase subunit 2 (COX2) as the mtDNA marker and ribosomal protein S18 (Rps18) as the nuclear reference gene for normalization. Primer sequences are listed in **Table S2.**

### Co-immunoprecipitation (CO-IP)

According to the manufacturer's instructions, Co-IP was performed using the Protein A/G Magnetic Bead IP Kit (P2179M, Beyotime, China). As described above, total protein was extracted. To determine the acetylation level of PGC-1α, the protein lysate was incubated overnight at 4 °C with Dynabeads conjugated to a PGC-1α primary antibody for immunoprecipitation. Subsequently, the immunoprecipitated complexes were collected and analyzed by western blotting.

### Statistical analysis

Results are expressed as mean ± standard deviation (SD), derived from at least three technical replicates per experiment. Data were normally distributed. Unpaired Student's t-tests were used for comparisons between two groups, while one-way or two-way analysis of variance (ANOVA) was applied for multigroup comparisons. Statistical significance was defined as P < 0.05. All statistical analyses and histogram generation were performed using GraphPad Prism software (version 9.0).

## Results

### Simulation of microgravity reduced Piezo1 expression and induced bone loss

**Figure 1:**
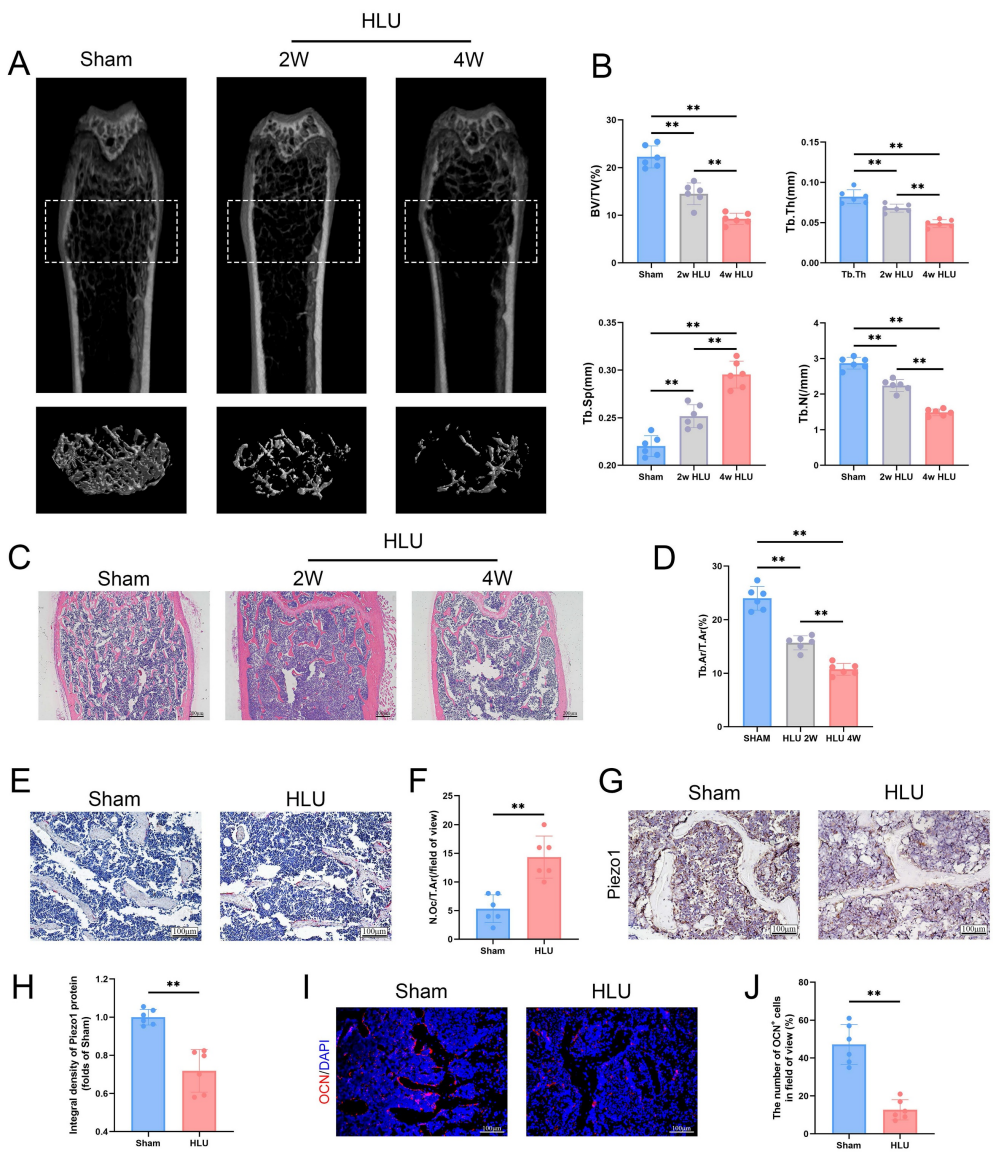
** Simulation of microgravity reduced Piezo1 expression and induced bone loss.** (A) Representative 3D micro-CT reconstructions of distal femoral trabecular architecture in sham and hindlimb-unloaded (HLU) mice (2 and 4 weeks). (B) Quantitative analysis of trabecular bone parameters: bone volume fraction (BV/TV), trabecular number (Tb.N), trabecular thickness (Tb.Th), and trabecular separation (Tb.Sp). Data: Mean ± SD, n = 6, *p< 0.05, **p < 0.01. (C) Representative HE-stained sections of distal femurs. Scale bar = 200 µm. (D) Quantitative analysis of B.Ar/T.Ar. B.Ar = bone area; T.Ar = total area. Data: Mean ± SD, n = 6, *p< 0.05, **p < 0.01. (E) Representative TRAP-stained sections showing osteoclasts. Scale bar = 100 µm. (F) Quantitative analysis of osteoclast density (N.Oc/T.Ar). *N.Oc* = number of osteoclasts. Data: Mean ± SD, n = 6, *p< 0.05, **p < 0.01. (G) Immunohistochemical staining of Piezo1 in femoral sections. Scale bar = 100 µm. (H) Quantitative analysis of Piezo1 expression level. Data: Mean ± SD, n = 6, *p< 0.05, **p < 0.01. (I) Representative immunofluorescence images of osteocalcin (OCN; red) in distal femurs at week 4. Scale bar = 100 µm. (J) The number of OCN-positive cells. Data: Mean ± SD, n = 6, *p< 0.05, **p < 0.01.

To study the effect of mechanical unloading on bone mass, we utilized the hind limb unloading (HLU) model to simulate the effects of microgravity on bone in vivo. Micro-CT analysis of the distal femur revealed significant trabecular bone loss in the HLU group compared to Sham group after 2 and 4 weeks (Figure 1A). Specifically, bone volume fraction (BV/TV), trabecular number (Tb.N), and trabecular thickness (Tb.Th) were significantly decreased, while trabecular separation (Tb.Sp) was significantly increased (Figure 1B). Based on these findings, 4 weeks was selected as the experimental endpoint. HE staining of the distal femur corroborated microgravity-induced bone loss, showing a significant reduction in the bone area/total area ratio (Figure 1C, D). TRAP staining showed a significant increase in osteoclast number in the HLU group compared to the Sham group (Figure 1E, F). Immunohistochemical (IHC) staining of femurs revealed a significant decrease of Piezo1 expression in the HLU group (Figure 1G, H). Osteocalcin (OCN) immunofluorescence indicated a significant reduction in OCN-positive osteoblasts within the trabecular bone area in the HLU group compared to Sham group (Figure 1I, J). Collectively, these results demonstrate that simulated microgravity significantly induces bone loss, characterized by reduced bone formation, increased bone resorption, and is associated with reduced Piezo1 expression.

### Piezo1 downregulation is associated with impaired osteogenesis and mitochondrial function in BMSCs under mechanical unloading

**Figure 2:**
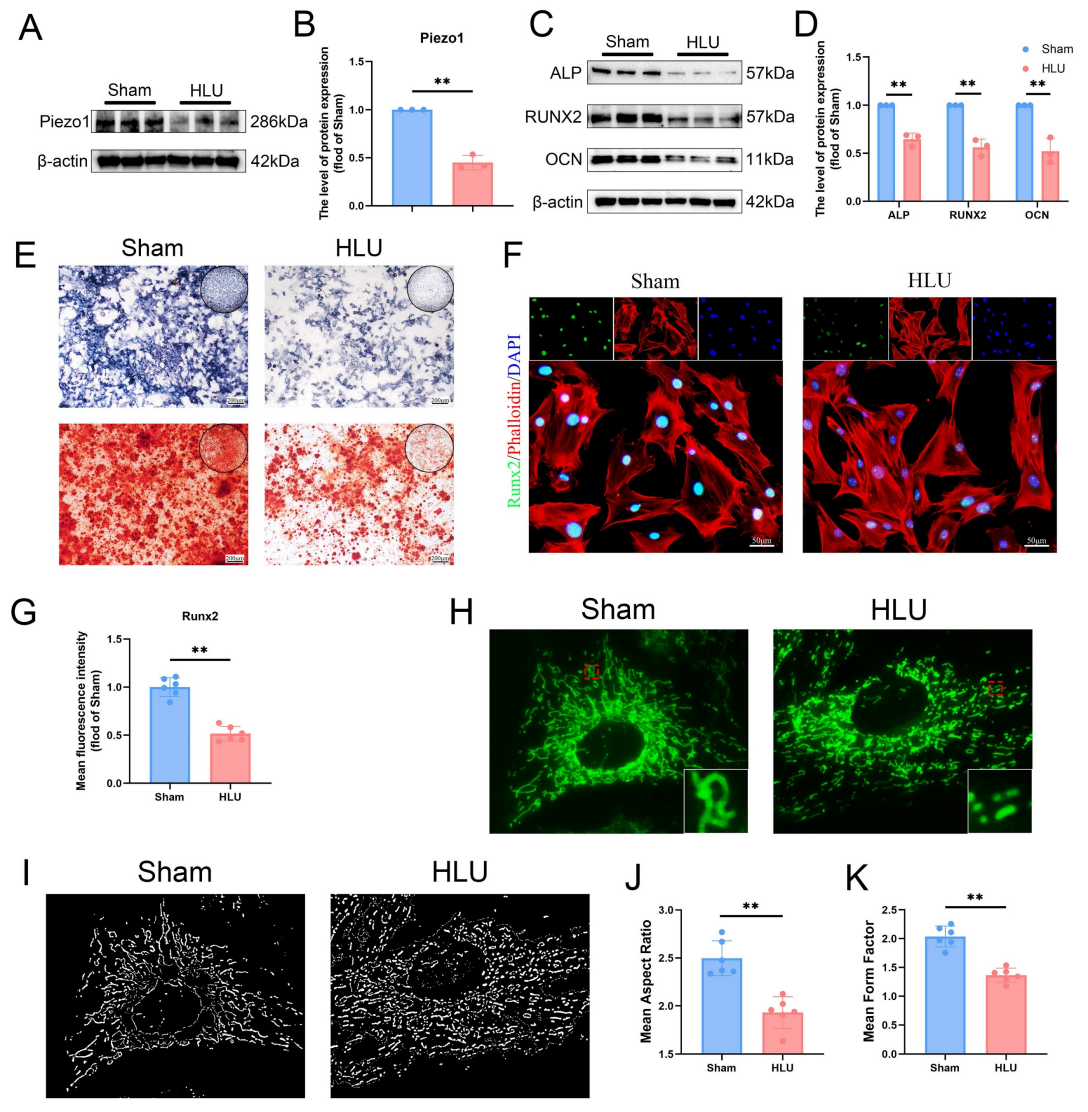
** Piezo1 downregulation is associated with impaired osteogenesis and mitochondrial function in BMSCs under mechanical unloading.** (A-B) Western blot analysis of Piezo1 protein expression levels. Mean ± SD, n = 3. *p < 0.05, **p < 0.01. (C-D) Western blot analysis of ALP, Runx2, and Ocn protein expression levels. Mean ± SD, n = 3. *p < 0.05, **p < 0.01. (E) Representative images alkaline phosphatase and alizarin red staining. Scale bar = 200 µm. (F-G) Representative immunofluorescence images and quantification analysis of Runx2. Scale bar = 50 µm. Mean ± SD, n = 6. *p < 0.05, **p < 0.01. (H-I) Representative images of MitoTracker staining (green) and 2D images. (J-K) Quantitative analysis of Mitochondrial morphology. Mean ± SD, n = 6. *p < 0.05, **p < 0.01.

To investigate the potential cellular mechanisms, we isolated bone marrow-derived mesenchymal stem cells (BMSCs) from the mice of HLU and Sham groups. Western blot analysis confirmed decreased Piezo1 expression in BMSCs from the HLU group (Figure 2A, B), accompanied by reduced levels of osteogenic marker proteins (ALP, RUNX2, OCN) following osteogenic induction (Figure 2C, D). Alkaline phosphatase (ALP) activity was higher in the Sham group than in the HLU group at day 7 of osteogenesis. By day 21, Alizarin Red S (ARS) staining revealed significantly greater mineralized matrix formation in the Sham group compared to the HLU group (Figure 2E). Immunofluorescence staining further demonstrated diminished nuclear expression of the osteogenic transcription factor RUNX2 in HLU group BMSCs (Figure 2F, G). These findings support an association between Piezo1 downregulation and impaired osteogenic differentiation in BMSCs under mechanical unloading. Given the reported link between Piezo1 and mitochondrial function^10^ and the critical role of mitochondria in osteogenesis^30^, we examined mitochondrial morphology using MitoTracker Green staining. Results showed that mitochondria in HLU group BMSCs exhibited increased fragmentation (Figure 2H, I). The analysis results of mitochondrial Analyzer software showed that the values of AR (Figure 2J) and FF (Figure 2K) were lower than those of Sham group. To investigate if mitochondrial dysfunction contributes to the impaired osteogenesis, we treated HLU BMSCs with the mitochondrial-targeted antioxidant Mitoquinone (MitoQ). MitoQ treatment partially rescued the expression of osteogenic markers (Figure S2A, B), as well as ALP activity and mineralized nodule formation (Figure S2C). Collectively, these data suggest that Piezo1 downregulation under mechanical unloading is associated with impaired osteogenic differentiation and mitochondrial dysfunction in BMSCs, and that ameliorating mitochondrial oxidative stress can partially rescue the osteogenic deficit.

### Piezo1 activation rescues osteogenesis and mitochondrial function in mechanically unloaded BMSCs

**Figure 3:**
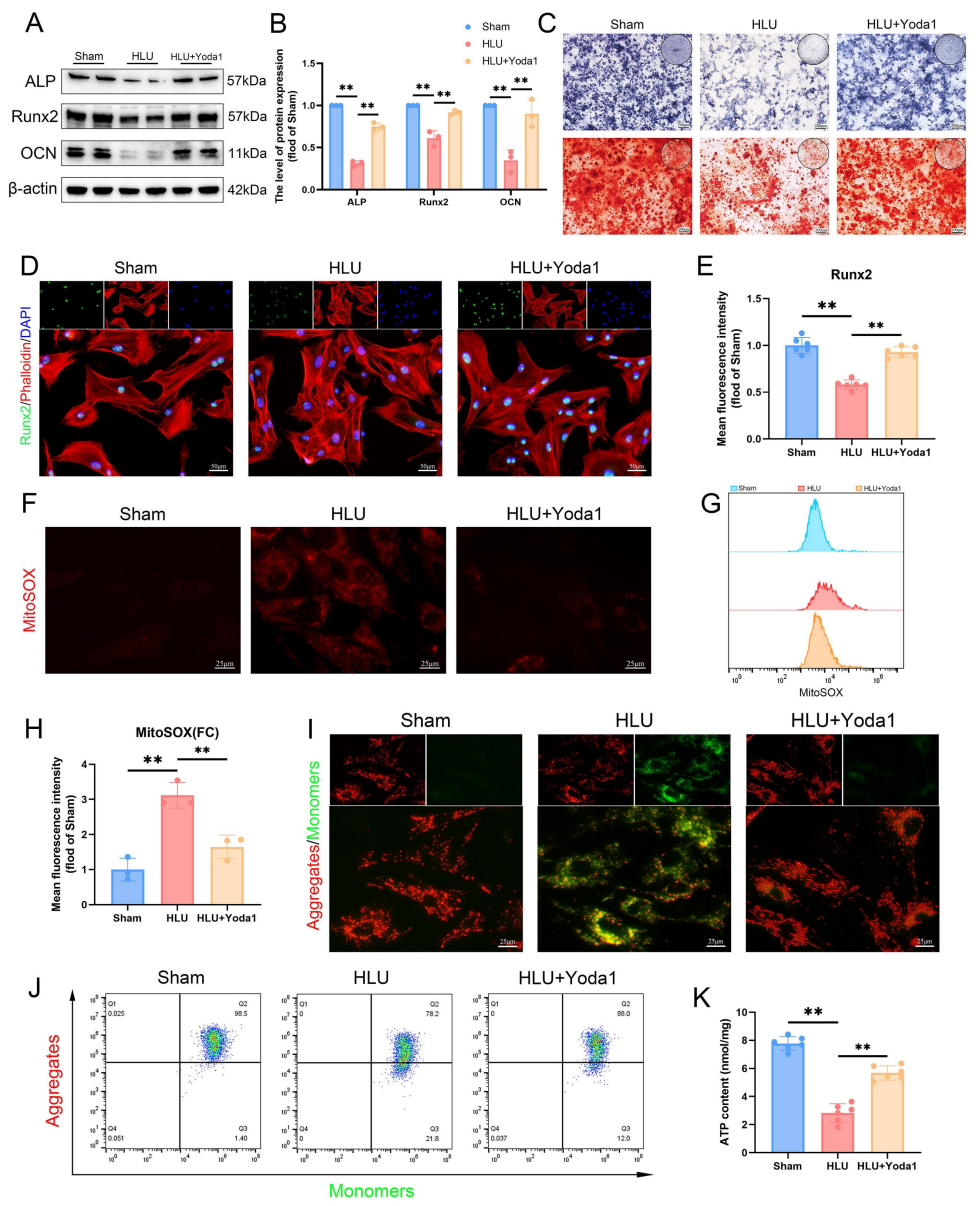
** Piezo1 activation rescues osteogenesis and mitochondrial function in mechanically unloaded BMSCs.** (A-B) Western blot analysis of ALP, Runx2, and Ocn protein expression levels. Mean ± SD, n = 3. *p < 0.05, **p < 0.01. (C) Representative images alkaline phosphatase and alizarin red staining. Scale bar = 200 µm. (D-E) Representative immunofluorescence images and quantification analysis of Runx2. Scale bar = 50 µm. Mean ± SD, n = 6. *p < 0.05, **p < 0.01. (F) Representative immunofluorescence images of mtROS. Scale bar = 25 µm. (G-H) Flow cytometrys and quantitative analysis of the mtROS in BMSCs of the indicated group. Mean ± SD, n = 3. *p < 0.05, **p < 0.01. (I) Representative images of JC-1 staining. Scale bar = 25 µm. (J) Flow cytometrys images of JC-1 staining. (K) Quantitative analysis of ATP level.

To further investigate the role of Piezo1 in mitochondrial function and osteogenesis under unloading, we treated BMSCs from tail-suspended mice with Yoda1, a Piezo1 agonist. Based on concentration-response experiments assessing cell proliferation (Figure S3) and literature reports^22^, we selected 5 μM Yoda1 for subsequent experiments. Yoda1 treatment increased levels of osteogenic proteins (ALP, RUNX2, OCN) compared to the untreated HLU group (Figure 3A, B). ALP activity and matrix mineralization were increased by Yoda1 treatment (Figure 3C). Additionally, immunofluorescence confirmed enhanced nuclear RUNX2 expression (Figure 3D, E). Assessment of mitochondrial function revealed that mechanical unloading increased mitochondrial reactive oxygen species (mtROS) accumulation (Figure 3F, G), decreased mitochondrial membrane potential (Δψm) (Figure 3I, J), and reduced ATP levels (Figure 3K) relative to the Sham group. Yoda1 treatment significantly ameliorated these mitochondrial impairments (Figure 3F-K). Collectively, these results indicate that pharmacological activation of Piezo1 by Yoda1 rescues osteogenic differentiation capacity and improves mitochondrial function in BMSCs subjected to mechanical unloading.

### Piezo1-mediated Ca²⁺ influx activates CaMKII and is required for AMPK/SIRT1 signaling in unloaded BMSCs

**Figure 4:**
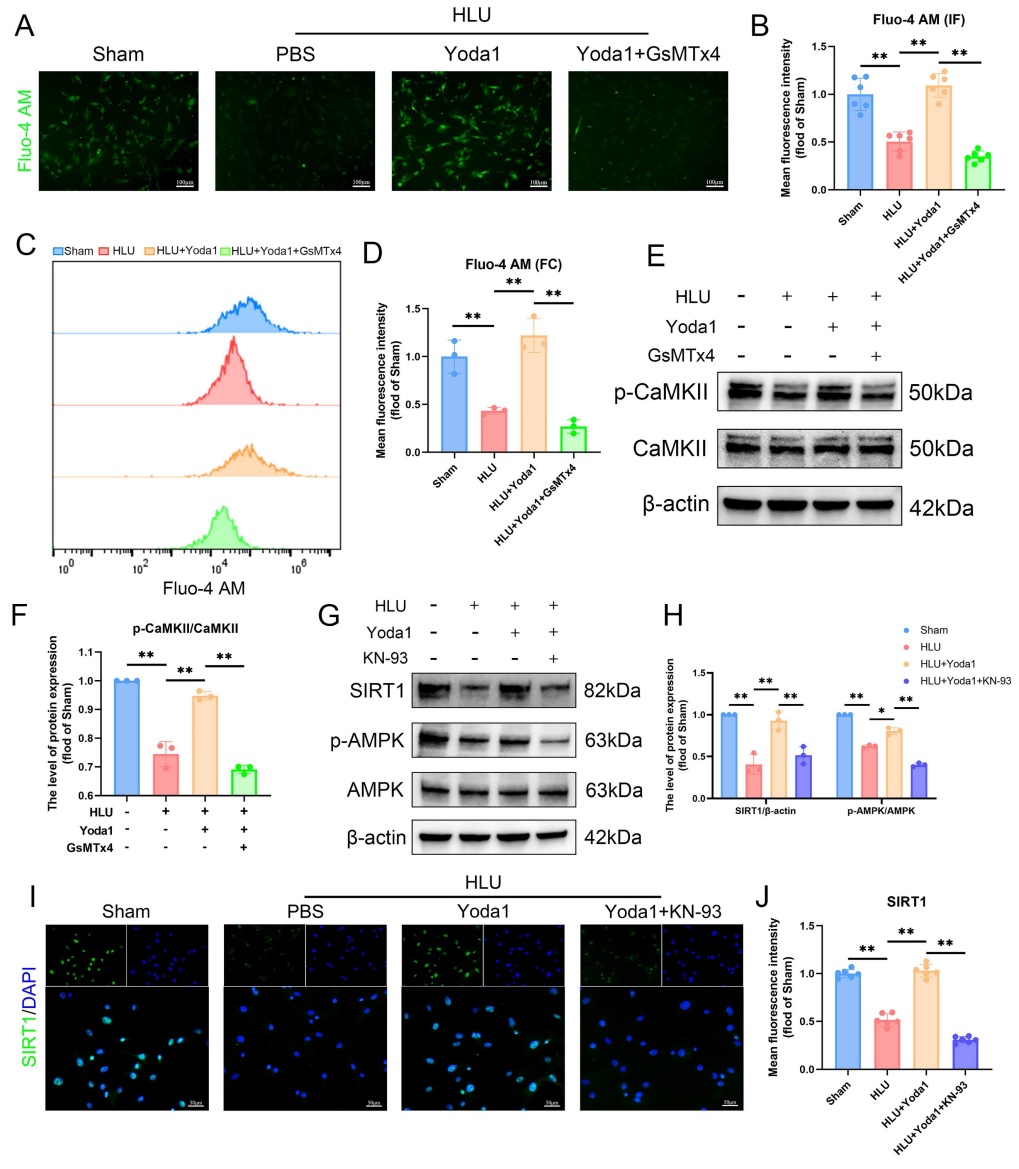
** Piezo1-mediated Ca²^+^ influx activates CaMKII and is required for AMPK/SIRT1 signaling in unloaded BMSCs.** (A-B) Representative immunofluorescence images and quantification analysis of Fluo-4 AM. Scale bar = 100µm. Mean ± SD, n = 6. *p < 0.05, **p < 0.01. (C-D) Flow cytometrys and quantitative analysis of the Fluo-4 AM in BMSCs. Mean ± SD, n = 3. *p < 0.05, **p < 0.01. (E-F) Western blot analysis of p-CaMKII, and CaMKII protein expression levels. Mean ± SD, n = 3. *p < 0.05, **p < 0.01. (G-H) Western blot analysis of SIRT1, p-AMPK, and AMPK protein expression levels. Mean ± SD, n = 3. *p < 0.05, **p < 0.01. (I-J) Representative immunofluorescence images and quantification analysis of SIRT1. Scale bar = 50µm. Mean ± SD, n = 6. *p < 0.05, **p < 0.01.

Piezo1 is a mechanically sensitive ion channel that promotes calcium ion influx upon activation. Using the Fluo-4 AM probe to detect intracellular calcium, fluorescence imaging revealed that Yoda1 treatment alleviated the reduced calcium influx caused by mechanical unloading (Figure 4A, B). Flow cytometry results also confirmed this effect (Figure 4C, D). This protective effect was blocked by pre-treatment with GsMTx4. Given the link between Ca²⁺ and Ca²⁺/calmodulin-dependent protein kinase II (CaMKII) activation, we examined this pathway. Western blot analysis showed that Yoda1 increased phosphorylated CaMKII (p-CaMKII) levels, and the effect was abolished by GsMTx4 pre-treatment (Figure 4E, F). Based on prior studies implicating downstream signaling involving CaMKII in BMSCs, we investigated the AMPK/SIRT1 pathway. Yoda1 treatment increased phospho-AMPK (p-AMPK) and SIRT1 protein levels compared to the HLU group (Figure 4G, H), and immunofluorescence confirmed increased nuclear SIRT1 expression (Figure 4I, J). Importantly, pre-treatment with the CaMKII inhibitor KN93 prevented the Yoda1-induced increases in p-AMPK and SIRT1 expression (Figure 4G-J). Collectively, these results indicate that Piezo1 activation triggers intracellular Ca²⁺ influx, leading to CaMKII activation, which is necessary for the subsequent upregulation of the AMPK/SIRT1 signaling pathway in mechanically unloaded BMSCs.

### Piezo1 activation promotes SIRT1-mediated deacetylation of PGC-1α in BMSCs

**Figure 5:**
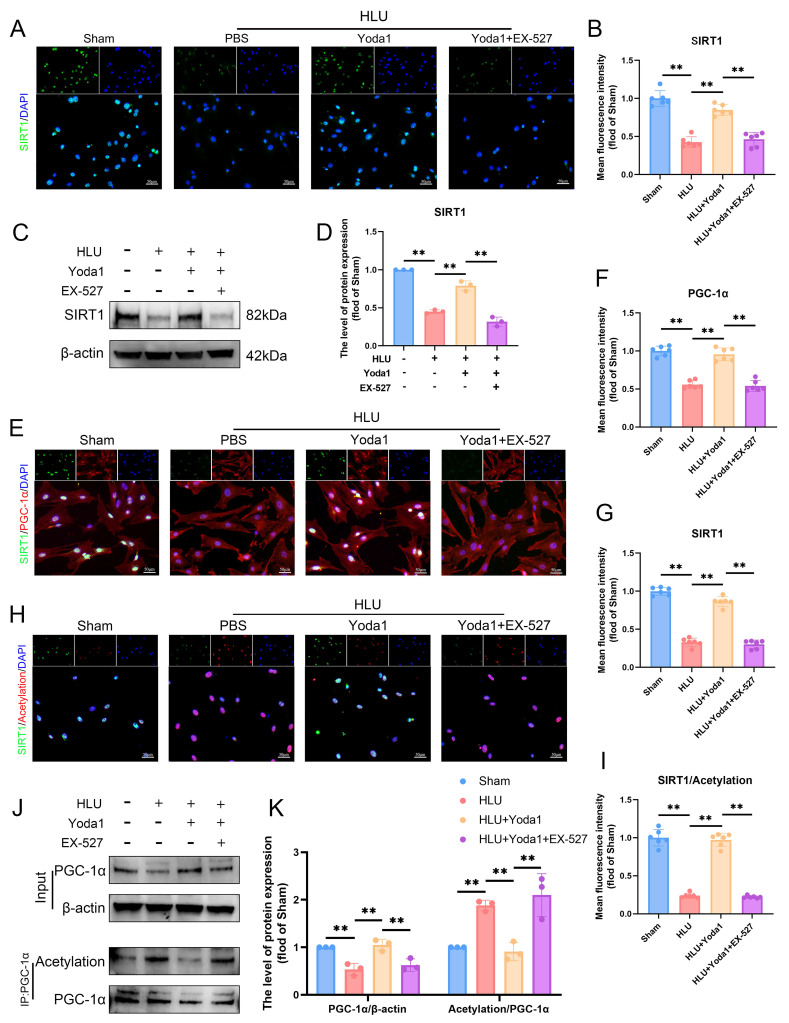
** Piezo1 activation promotes SIRT1-mediated deacetylation of PGC-1α in BMSCs.** (A-B) Representative immunofluorescence images and quantification analysis of SIRT1. Scale bar = 50µm. Mean ± SD, n = 6. *p < 0.05, **p < 0.01. (C-D) Western blot analysis of SIRT1 protein expression levels. Mean ± SD, n = 3. *p < 0.05, **p < 0.01. (E-G) Representative immunofluorescence images and quantification analysis of SIRT1, and PGC-1α. Scale bar = 50µm. Mean ± SD, n = 6. *p < 0.05, **p < 0.01. (H-I) Representative immunofluorescence images and quantification analysis of SIRT1/Acetylation. Scale bar = 50µm. Mean ± SD, n = 6. *p < 0.05, **p < 0.01. (J-K) Western blot analysis of PGC-1α protein and acetylation of PGC-1α expression levels. Mean ± SD, n = 3. *p < 0.05, **p < 0.01.

Peroxisome proliferator-activated receptor γ coactivator 1-α (PGC-1α) is a key regulator of mitochondrial biogenesis whose activity is modulated by acetylation status. SIRT1, as an important deacetylase, plays a crucial role in this process. To directly assess its regulation of PGC-1α acetylation, we conducted experiments using the specific SIRT1 inhibitor EX-527. EX-527 attenuated the Yoda1-induced increase in SIRT1 expression (Figure 5A-D). Immunofluorescence analysis revealed decreased SIRT1 expression in HLU group BMSCs, concomitant with reduced PGC-1α levels and increased overall nuclear protein acetylation (Figure 5E, H). Co-immunoprecipitation (Co-IP) assays specifically targeting PGC-1α confirmed that unloading downregulated PGC-1α protein expression while increasing its acetylation level (Figure 5J, K). Yoda1 treatment significantly enhanced SIRT1 expression and activity, thereby reducing the acetylation level of PGC-1α. Experimental data demonstrated that EX-527 can block Yoda1-induced deacetylation of PGC-1α (Figure 5E-K). Collectively, these findings demonstrate that Piezo1 activation promotes SIRT1 expression and activity, which in turn deacetylates PGC-1α in BMSCs under mechanical unloading conditions.

### PGC-1α deacetylation promotes TFAM-mediated mtDNA biogenesis and protects against apoptosis in unloaded BMSCs

**Figure 6:**
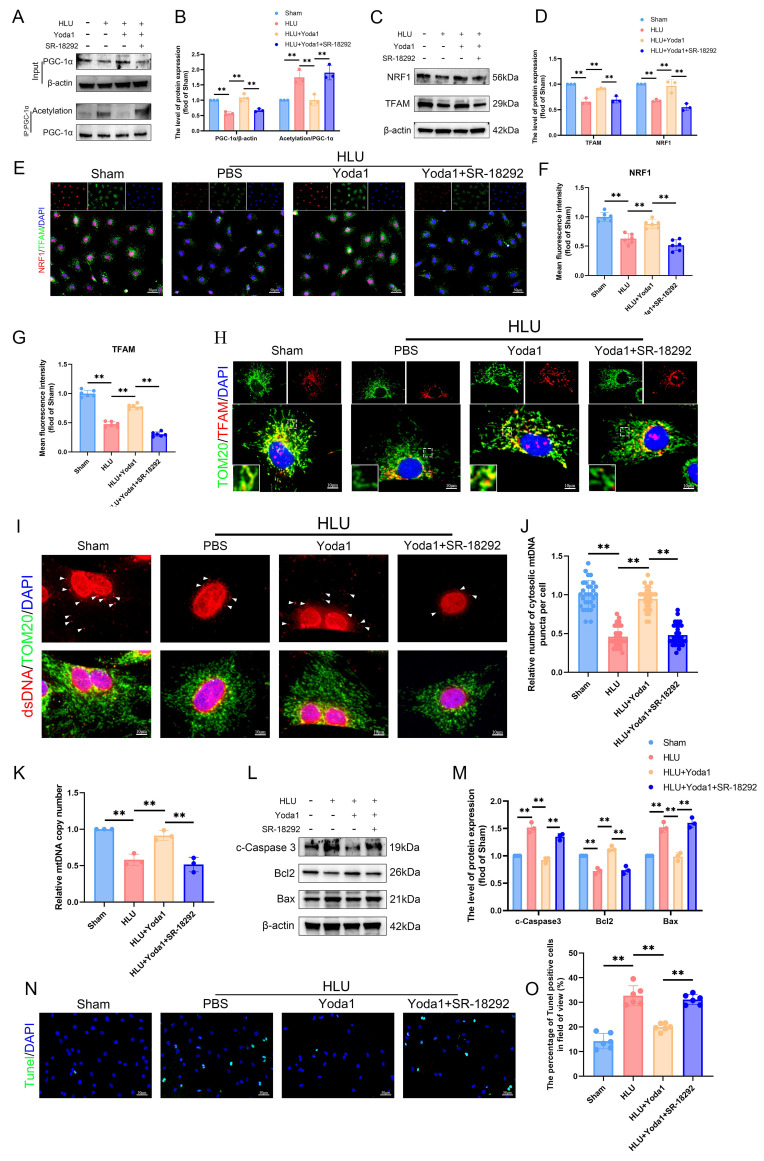
** PGC-1α deacetylation promotes TFAM-mediated mtDNA biogenesis and protects against apoptosis in unloaded BMSCs.** (A-B) Western blot analysis of PGC-1α protein and acetylation of PGC-1α expression levels. Mean ± SD, n = 3. *p < 0.05, **p < 0.01. (C-D) Western blot analysis of NRF1, and TFAM protein expression levels. Mean ± SD, n = 3. *p < 0.05, **p < 0.01. (E-G) Representative immunofluorescence images and quantification analysis of NRF1, and TFAM. Scale bar = 50µm. Mean ± SD, n = 6. *p < 0.05, **p < 0.01. (H) Representative immunofluorescence images of TOM20, and TFAM. Scale bar = 10µm. (I-J) Representative immunofluorescence images and quantification analysis of dsDNA. Scale bar = 10µm. Mean ± SD, n = 30. *p < 0.05, **p < 0.01. (K) Quantitative analysis of relative mtDNA copy number. Mean ± SD, n = 3. *p < 0.05, **p < 0.01. (L-M) Western blot analysis of c-Caspase3, Bcl2, and Bax protein expression levels. Mean ± SD, n = 3. *p < 0.05, **p < 0.01. (N-O) Representative immunofluorescence images and quantification analysis of Tunel-positive cells, and TFAM. Scale bar = 50µm. Mean ± SD, n = 6. *p < 0.05, **p < 0.01.

To further evaluate the effects of PGC-1α deacetylation on mitochondrial function regulation, we used SR-18292, a reported inhibitor of PGC-1α deacetylation. SR-18292 significantly attenuated the Yoda1-induced PGC-1α deacetylation (Figure 6A, B). Mitochondrial transcription factor A (TFAM) is essential for mitochondrial DNA (mtDNA) maintenance and transcription. Western blot analysis revealed significantly reduced expression of nuclear respiratory factor 1 (NRF1) and TFAM in HLU group BMSCs compared to sham controls (Figure 6C, D), confirmed by immunofluorescence (Figure 6E-G). Yoda1 treatment elevated NRF1 and TFAM levels (Figure 6C-G). Immunofluorescence demonstrated TFAM co-localization with the mitochondrial marker TOM20. Mitochondrial TFAM abundance was lower in HLU-group BMSCs, and Yoda1 treatment partially restored it (Figure 6H). Using an anti-dsDNA antibody, we observed fewer cytoplasmic mitochondrial DNA (mtDNA) puncta in HLU-group BMSCs, correlating with TFAM reduction. Yoda1 rescued mtDNA puncta formation (Figure 6I, J). Furthermore, quantitative PCR confirmed a significant reduction in mtDNA copy number in the HLU group, which was reversed by Yoda1 (Figure 6K). All of the above effects of Yoda1 were blocked by SR-18292. Given the link between mitochondrial dysfunction and apoptosis, and the observed mtDNA depletion, we analyzed apoptosis. Western blot showed increased levels of cleaved caspase-3 and Bax/Bcl-2 ratio in the HLU group, indicative of enhanced apoptosis (Figure 6L, M). TUNEL staining confirmed increased apoptotic cells (Figure 6N, O). Yoda1 treatment reduced these apoptotic markers. Importantly, SR-18292 blocked the anti-apoptotic effect of Yoda1 (Figure 6L-O). These results indicate that Piezo1 activation, via promoting SIRT1-mediated deacetylation of PGC-1α, enhances NRF1/TFAM expression, mtDNA biogenesis, and mitochondrial function.

### The AMPK/SIRT1/PGC-1α axis mediates Piezo1's effects on mitochondrial function and osteogenesis in BMSCs

**Figure 7:**
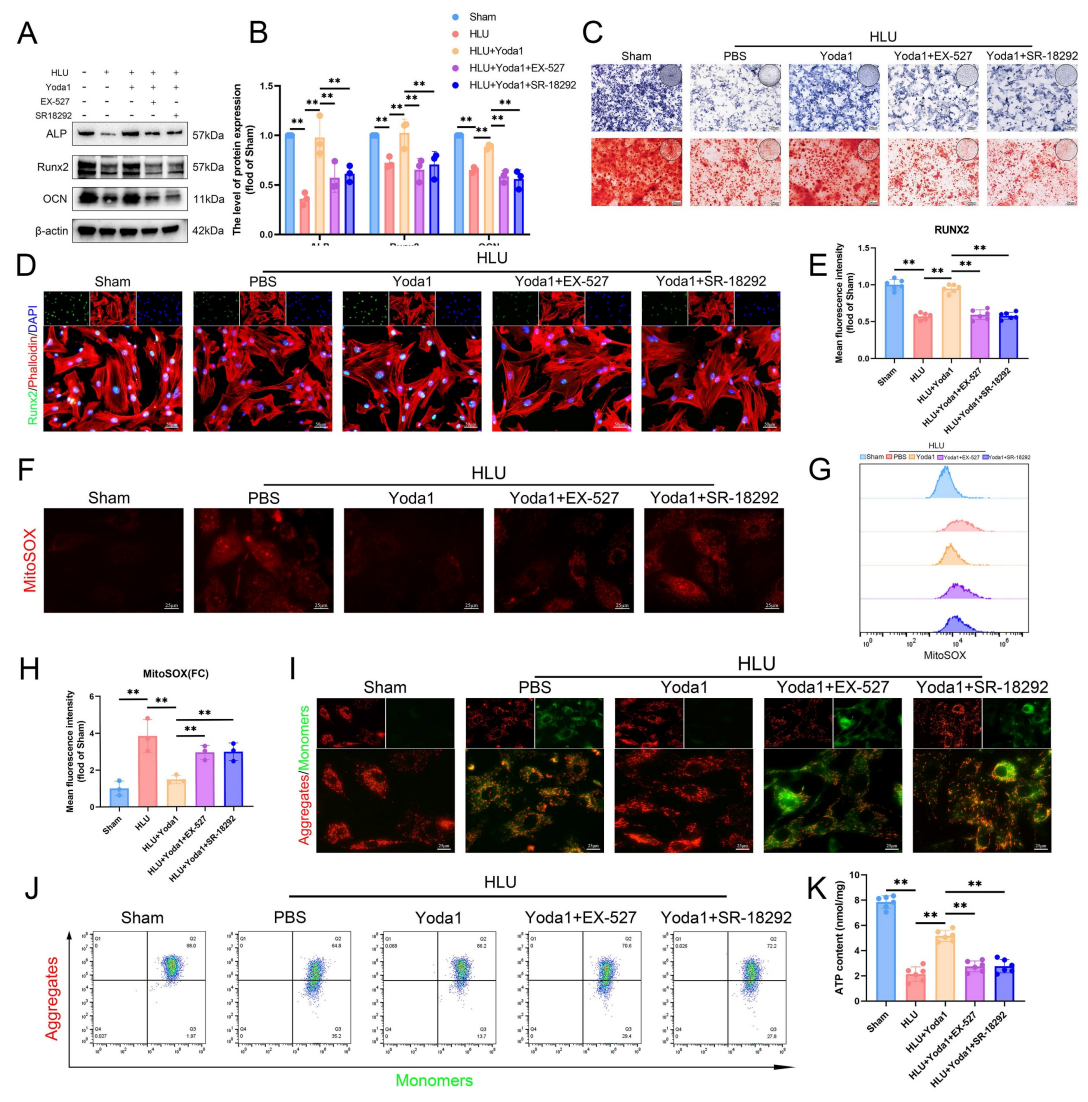
** The AMPK/SIRT1/PGC-1α axis mediates Piezo1's effects on mitochondrial function and osteogenesis in BMSCs.** (A-B) Western blot analysis of ALP, Runx2, and Ocn protein expression levels. Mean ± SD, n = 3. *p < 0.05, **p < 0.01. (C) Representative images alkaline phosphatase and alizarin red staining. (D and E) Representative immunofluorescence images and quantification analysis of Runx2. Scale bar = 50 µm. Mean ± SD, n = 6. *p < 0.05, **p < 0.01. (F) Representative immunofluorescence images of mtROS. Scale bar = 25 µm. (G-H) Flow cytometrys and quantitative analysis of the mtROS in BMSCs of the indicated group. Mean ± SD, n = 3. *p < 0.05, **p < 0.01. (I) Representative images of JC-1 staining. Scale bar = 25 µm. (J) Flow cytometrys images of JC-1 staining. (K) Quantitative analysis of ATP level.

To determine if the AMPK/SIRT1/PGC-1α pathway is necessary for Piezo1-mediated improvements in mitochondrial function and osteogenesis, we pre-treated HLU group BMSCs with EX-527 (SIRT1 inhibitor) or SR-18292 (PGC-1α deacetylation inhibitor) followed by Yoda1. Both inhibitors attenuated the Yoda1-induced upregulation of osteogenic proteins (ALP, RUNX2, OCN) (Figure 7A, B), ALP activity, and matrix mineralization (Figure 7C). Immunofluorescence showed reduced nuclear RUNX2 expression (Figure 7D, E). Similarly, both inhibitors blocked Yoda1's improvement of mitochondrial function, as evidenced by reversal of the decrease in mtROS (Figure 7F-H), prevention of the recovery of Δψm (Figure 7I, J), and inhibition of the increase in ATP levels (Figure 7K). In summary, these findings indicate that the AMPK/SIRT1/PGC-1α signaling axis is essential for mediating the beneficial effects of Piezo1 activation on mitochondrial function and osteogenic differentiation in BMSCs of mechanical unloading.

### Piezo1 activation attenuates HLU-induced bone loss in a SIRT1-dependent manner

**Figure 8:**
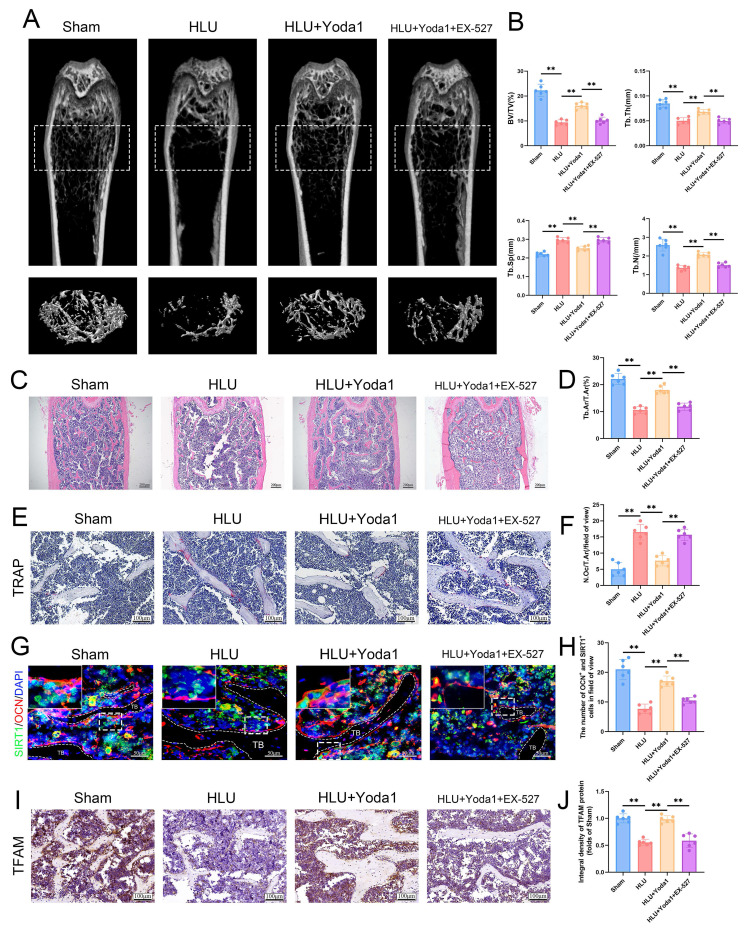
** Piezo1 activation attenuates HLU-induced bone loss in a SIRT1-dependent manner.** (A) Representative 3D micro-CT reconstructions of distal femoral trabecular architecture. (B) Quantitative analysis of trabecular bone parameters: bone volume fraction (BV/TV), trabecular number (Tb.N), trabecular thickness (Tb.Th), and trabecular separation (Tb.Sp). Data: Mean ± SD, n = 6, *p< 0.05, **p < 0.01. (C) Representative HE-stained sections of distal femurs. Scale bar = 100 µm. (D) Quantitative analysis of B.Ar/T.Ar. B.Ar = bone area; T.Ar = total area. Data: Mean ± SD, n = 6, *p< 0.05, **p < 0.01. (E) Representative TRAP-stained sections showing osteoclasts. Scale bar = 100 µm. (F) Quantitative analysis of osteoclast density (N.Oc/T.Ar). N.Oc = number of osteoclasts. Data: Mean ± SD, n = 6, *p< 0.05, **p < 0.01. (G) Representative immunofluorescence images of osteocalcin (OCN; red) in distal femurs at week 4. Scale bar = 100 µm. (H) The number of OCN and SIRT-positive cells. Data: Mean ± SD, n = 6, *p< 0.05, **p < 0.01. (I) Immunohistochemical staining of TFAM in femoral sections. Scale bar = 100 µm. (J) Quantitative analysis of TFAM expression level. Data: Mean ± SD, n = 6, *p< 0.05, **p < 0.01.

To ultimately validate this finding in an in vivo setting, we assessed the effect of Yoda1 in HLU mice. Systemic administration of Yoda1 significantly attenuated unloading-induced bone loss. Micro-CT analysis showed increased BV/TV, Tb.N, Tb.Th, and decreased Tb.Sp in Yoda1-treated HLU mice compared to the untreated HLU mice (Figure S4A, B). HE staining further confirmed the protective effect of Yoda1 on bone structure (Figure S4C, D). The 5 mg/kg dose was chosen as the ultimate dose for subsequent mechanistic investigations due to its stronger effect. Histological analysis of major organs confirmed that the 5 mg/kg dose exhibited no toxic effects in mice (Figure S5). To investigate the requirement of SIRT1 for Yoda1's osteogenic effects in vivo, HLU mice received intraperitoneal injections of the SIRT1 inhibitor EX-527 prior to Yoda1 treatment. EX-527 abolished the beneficial effects of Yoda1 on trabecular bone parameters (BV/TV, Tb.N, Tb.Th, Tb.Sp) as measured by micro-CT (Figure 8A, B). The results of the HE staining also supported the same conclusion (Figure 8C, D). TRAP staining revealed a reduction in osteoclast numbers following Yoda1 administration, but this effect was also suppressed by EX-527 (Figure 8E, F). Furthermore, the SIRT1/osteocalcin (OCN) co-immunofluorescence demonstrated an increase in SIRT1 and OCN dual-positive osteoblasts in the Yoda1-treated group relative to the HLU group. This increase in dual-positive osteoblasts was suppressed by EX-527 co-treatment (Figure 8G, H). Meanwhile, to further validate the downstream mechanism, we performed TFAM immunohistochemistry and showed that Yoda1 promoted TFAM expression, which was inhibited by EX-527 (Figure 8I, J). Collectively, these in vivo results demonstrate that SIRT1 activity is required for Yoda1 to exert its protective effects against microgravity-induced bone loss and to promote osteoblast activity.

## Discussion

Mechanical stimulation is widely recognized as a critical factor in bone formation, development, and repair. The primary cause of disuse osteoporosis (DOP) lies in the absence of adequate mechanical stimulation, which can disrupt bone remodeling homeostasis^31^. Recently, piezoelectric ion channels (Piezo1 and Piezo2) have been identified as mechanically sensitive ion channels and play a key role in various physiological processes^32^. Piezo1 is predominantly expressed in non-sensory tissues and non-neuronal cells, where it detects diverse mechanical stimuli, such as static pressure, shear stress, and tensile force^9^, ^33^. Furthermore, Piezo1 serves as a critical regulator of bone remodeling by modulating osteoblast lineage cells and osteoclasts in osteoporosis^9^. Piezo1 plays a crucial role in the mechanosensitive differentiation of mesenchymal stem cells (MSCs)^34^, ^35^. Bone marrow mesenchymal stem cells (BMSCs), as the principal osteogenic precursors implicated in osteoporosis^36^, exhibit significantly reduced Piezo1 expression under mechanical unloading^2^. Our findings confirm that mechanical unloading suppresses Piezo1 expression and osteogenic differentiation in BMSCs, mirroring the pathognomonic features of DOP. Importantly, we establish a direct mechanistic connection by linking the loss of mechanical stimulation to suppressed Piezo1 activity as the underlying cause of the osteogenic defect.

Mitochondria serve as the powerhouse of the cell, producing ATP through oxidative phosphorylation^37^. Moreover, they play a key role in regulating the osteogenic differentiation of BMSCs^36^, ^38^. Mechanical signals regulate mitochondrial structure and enhance mitochondrial function in diverse cell types^39^, ^40^. Studies demonstrate that Piezo1 regulates mitochondrial function and affects the functions of osteoblasts and mesenchymal stem cells^22^, ^41^. In line with these observations, our study revealed that mechanical unloading in mice led to significant mitochondrial dysfunction in BMSCs, which was accompanied by a marked decline in osteogenic differentiation capacity. Notably, we further demonstrated that pharmacological activation of Piezo1 using Yoda1 effectively restored mitochondrial function and promoted osteogenic differentiation under unloading conditions. These findings not only corroborate earlier reports on the mechanosensitive regulation of mitochondria, but also establish a direct functional link between Piezo1 activation and the recovery of mitochondrial integrity in BMSCs subjected to mechanical deprivation.

The activation of Piezo1 leads to a sharp increase in cytoplasmic calcium^42^. CaMKII, a serine/threonine kinase, mediates the second messenger effects of Ca²⁺ in cells. It also participates in various Piezo1-mediated biological processes^25^, ^43^. Piezo1-mediated calcium influx activated the CaMKII/CREB pathway and enhanced osteogenesis in BMSCs of senile osteoporosis^43^. We found that Piezo1 activation robustly enhanced intracellular calcium flux and significantly increased the levels of phosphorylated CaMKII (p-CaMKII) in BMSCs. Conversely, inhibiting mechanosensitive calcium entry with GsMTx4 effectively suppressed p-CaMKII levels. These results mechanistically connect Piezo1 activation to the CaMKII pathway in the context of disuse osteoporosis.

Adenosine 5'-monophosphate (AMP)-activated protein kinase (AMPK) is a heterotetrameric complex comprising α, β, and γ subunits. It serves as an essential regulator of cellular energy metabolism^44^, ^45^. Activation of Piezo1 modulated osteoblastic differentiation via the AMPK-autophagy signaling axis^12^. SIRT1, a NAD+-dependent histone deacetylase, regulates diverse cellular processes such as proliferation, apoptosis, autophagy, and senescence. And it can be affected by the level of phosphorylated AMPK^46^. In the skeletal system, SIRT1 has emerged as a pivotal positive regulator of bone homeostasis^47^. It promotes the osteogenic differentiation of BMSCs and inhibits osteoclastogenesis^48^. The activity and expression of SIRT1 are regulated by mechanical stimulation. The study has shown that mechanical stimulation can enhance the expression of SIRT1 in BMSCs, thereby promoting autophagy and promoting the differentiation process of osteoblasts^49^. To systematically dissect the hierarchical relationship within this signaling network, we investigated whether the Piezo1-CaMKII axis, positioned upstream, governs the activation of AMPK and SIRT1 under mechanical unloading. Our results revealed that Yoda1-induced activation of Piezo1 significantly upregulated the levels of phosphorylated AMPK (p-AMPK) and SIRT1. Crucially, this effect was completely abolished by the competitive CaMKII inhibitor KN-93. This finding not only confirms that Piezo1-triggered calcium influx and subsequent CaMKII activation are prerequisites for modulating the AMPK/SIRT1 pathway in this context, but also identifies a novel linear signaling cascade in the mechanotransduction of BMSCs under disuse conditions, where the signal flows through Piezo1/Ca²⁺/CaMKII/AMPK/SIRT1.

As the most prominent downstream target of Sirt1, peroxisome proliferator-activated receptor γ coactivator 1-α (PGC-1α) plays a vital role in regulating mitochondrial function and serves as a key regulator of bone homeostasis^50^, ^51^. It is a pivotal regulator of cell fate, whose expression declines during aging in both human and mouse skeletal stem cells (SSCs). Loss of PGC-1α in SSCs impairs bone formation capacity^52^. The activity of PGC-1α is closely linked to its acetylation status^53^, ^54^. Using co-immunoprecipitation (CO-IP), we observed increased acetylation of PGC-1α in BMSCs from tail-suspended mice, which was associated with reduced PGC-1α activity. AMPK has been shown to activate PGC-1α, in part, by increasing NAD+ levels, thereby stimulating SIRT1-mediated deacetylation of PGC-1α^55^. Consistent with this, we found that Piezo1-mediated activation of the AMPK/SIRT1 pathway promoted PGC-1α expression and reduced its acetylation. Inhibition of SIRT1 activity led to a significant decrease in PGC-1α expression and a corresponding increase in its acetylation, which would be expected to further diminish its activity. These results link the mechanical transduction induced by Piezo1 to the reversible acetylation control of PGC-1α under mechanical unloading.

Mitochondrial biogenesis is essential for maintaining mitochondrial function and cellular homeostasis under diverse pathological conditions, and plays a crucial role in the osteogenic function of BMSCs^56^, ^57^. Mitochondrial biogenesis is orchestrated by PGC-1α, which activates nuclear respiratory factor (NRF1) expression, leading to the activation of mitochondrial transcription factor A (TFAM) and subsequent mtDNA replication^17^. Our results showed that enhanced PGC-1α deacetylation increased mitochondrial DNA (mtDNA) copy number, augments mitochondrial function, and elevated ATP levels, concurrent with increased expression of TFAM and NRF1. Conversely, inhibition of PGC-1α deacetylation blocked the Piezo1-mediated increases in TFAM, NRF1, mtDNA copy number, mitochondrial function, and ATP levels. This demonstrates that SIRT1-dependent deacetylation and activation of PGC-1α are indispensable for Piezo1-driven mitochondrial biogenesis. These findings confirm the pivotal role of PGC-1α deacetylation in driving mitochondrial biogenesis. More significantly, they establish Piezo1-mediated mechanosignaling as a novel upstream regulator of this process.

In summary, this study demonstrates that Piezo1 activation promotes mitochondrial biogenesis by promoting AMPK/SIRT1-mediated deacetylation and activation of PGC-1α, thereby enhancing the osteogenic differentiation of BMSCs and mitigating bone loss induced by mechanical unloading. While this study delineates the Piezo1-mediated signaling pathway in disuse osteoporosis, several limitations should be acknowledged. Firstly, although the absence of acute toxicity was observed in our short-term model, the therapeutic potential of systemic Yoda1 administration is still constrained by its non-specific activation of Piezo1 channels in non-skeletal tissues, which raises concerns about potential off-target effects. Future therapies would benefit from developing bone-targeted delivery systems to localize Piezo1 activation. Secondly, the crucial mechanistic insights provided by pharmacological inhibitors must be interpreted with consideration of their inherent limitations, such as potential off-target effects and incomplete target inhibition. Future studies employing conditional knockout models or RNA interference approaches will be essential to validate these findings with greater genetic specificity and establish definitive causal relationships.

## Conclusion

Based on this study, mechanical unloading in disuse osteoporosis downregulates Piezo1 in bone marrow mesenchymal stem cells (BMSCs), impairing calcium influx and disrupting the AMPK/SIRT1 pathway. This leads to PGC-1α hyperacetylation, mitochondrial dysfunction, and reduced osteogenic differentiation. Pharmacological Piezo1 activation partially restores mitochondrial function and bone formation via SIRT1-mediated PGC-1α deacetylation, identifying Piezo1 as a key mechanosensor and promising target for treatment.

## Supplementary Material

Supplementary figures and tables.

## Abbreviations

DOP: Disuse Osteoporosis
BMSCs: Bone Marrow Mesenchymal Stem Cells
FSS: Fluid Shear Stress
HP: Hydrostatic Pressure
HLU: Hindlimb Unloading
Micro-CT: Micro-Computed Tomography
BV/TV: Bone Volume/Total Volume
Tb.N: Trabecular Number
Tb.Th: Trabecular Thickness
Tb.Sp: Trabecular Separation
IHC: Immunohistochemistry
OCN: Osteocalcin
ALP: Alkaline Phosphatase
ARS: Alizarin Red S
Δψm: Mitochondrial Membrane Potential
mtROS: Mitochondrial Reactive Oxygen Species
SIRT1: Sirtuin 1
PGC-1α: Peroxisome Proliferator-Activated Receptor Gamma Coactivator 1-Alpha
AMPK: AMP-Activated Protein Kinase
CO-IP: Co-Immunoprecipitation
TFAM: Mitochondrial Transcription Factor A
NRF1: Nuclear Respiratory Factor 1

## References

[1] Qin L, Liu W, Cao H, Xiao G (2020). Molecular mechanosensors in osteocytes. Bone research.

[2] Hu Y, Tian H, Chen W, Liu Y, Cao Y, Pei H (2023). The Critical Role of The Piezo1/β-catenin/ATF4 Axis on The Stemness of Gli1(+) BMSCs During Simulated Microgravity-Induced Bone Loss. Advanced science (Weinheim, Baden-Wurttemberg, Germany).

[3] Li L, Zhang C, Chen JL, Hong FF, Chen P, Wang JF (2019). Effects of simulated microgravity on the expression profiles of RNA during osteogenic differentiation of human bone marrow mesenchymal stem cells. Cell proliferation.

[4] Nabavi N, Khandani A, Camirand A, Harrison RE (2011). Effects of microgravity on osteoclast bone resorption and osteoblast cytoskeletal organization and adhesion. Bone.

[5] Henstock JR, Rotherham M, Rashidi H, Shakesheff KM, El Haj AJ (2014). Remotely Activated Mechanotransduction via Magnetic Nanoparticles Promotes Mineralization Synergistically With Bone Morphogenetic Protein 2: Applications for Injectable Cell Therapy. Stem cells translational medicine.

[6] Vining KH, Mooney DJ (2017). Mechanical forces direct stem cell behaviour in development and regeneration. Nature reviews Molecular cell biology.

[7] Kreke MR, Huckle WR, Goldstein AS (2005). Fluid flow stimulates expression of osteopontin and bone sialoprotein by bone marrow stromal cells in a temporally dependent manner. Bone.

[8] Sugimoto A, Miyazaki A, Kawarabayashi K, Shono M, Akazawa Y, Hasegawa T (2017). Piezo type mechanosensitive ion channel component 1 functions as a regulator of the cell fate determination of mesenchymal stem cells. Scientific reports.

[9] Wang L, You X, Lotinun S, Zhang L, Wu N, Zou W (2020). Mechanical sensing protein PIEZO1 regulates bone homeostasis via osteoblast-osteoclast crosstalk. Nature communications.

[10] Bahat A, Gross A (2019). Mitochondrial plasticity in cell fate regulation. The Journal of biological chemistry.

[11] Michaletti A, Gioia M, Tarantino U, Zolla L (2017). Effects of microgravity on osteoblast mitochondria: a proteomic and metabolomics profile. Scientific reports.

[12] Wu Y, Xu X, Liu F, Jing Z, Shen D, He P (2023). Three-Dimensional Matrix Stiffness Activates the Piezo1-AMPK-Autophagy Axis to Regulate the Cellular Osteogenic Differentiation. ACS biomaterials science & engineering.

[13] Xu W, Yan J, Ocak U, Lenahan C, Shao A, Tang J (2021). Melanocortin 1 receptor attenuates early brain injury following subarachnoid hemorrhage by controlling mitochondrial metabolism via AMPK/SIRT1/PGC-1α pathway in rats. Theranostics.

[14] Liu J, Wang J, Wang Z, Ren H, Zhang Z, Fu Y (2024). PGC-1α/LDHA signaling facilitates glycolysis initiation to regulate mechanically induced bone remodeling under inflammatory microenvironment. Bone.

[15] Shen Y, Wu L, Qin D, Xia Y, Zhou Z, Zhang X (2018). Carbon black suppresses the osteogenesis of mesenchymal stem cells: the role of mitochondria. Particle and fibre toxicology.

[16] Kaarniranta K, Kajdanek J, Morawiec J, Pawlowska E, Blasiak J (2018). PGC-1α Protects RPE Cells of the Aging Retina against Oxidative Stress-Induced Degeneration through the Regulation of Senescence and Mitochondrial Quality Control. The Significance for AMD Pathogenesis. International journal of molecular sciences.

[17] Wood Dos Santos T, Cristina Pereira Q, Teixeira L, Gambero A, J AV, Lima Ribeiro M (2018). Effects of Polyphenols on Thermogenesis and Mitochondrial Biogenesis. International journal of molecular sciences.

[18] Na J, Yang Z, Shi Q, Li C, Liu Y, Song Y (2024). Extracellular matrix stiffness as an energy metabolism regulator drives osteogenic differentiation in mesenchymal stem cells. Bioactive materials.

[19] Chu T, Wasi M, Guerra RM, Song X, Wang S, Sims-Mourtada J (2025). Skeletal response to Yoda1 and whole-body vibration in mice varied with animal age, bone compartment, treatment duration, and radiation exposure. Bone.

[20] Wasi M, Chu T, Guerra RM, Kooker R, Maldonado K, Li X (2024). Mitigating aging and doxorubicin induced bone loss in mature mice via mechanobiology based treatments. Bone.

[21] Li HR, Liu Q, Zhu CL, Sun XY, Sun CY, Yu CM (2023). β-Nicotinamide mononucleotide activates NAD+/SIRT1 pathway and attenuates inflammatory and oxidative responses in the hippocampus regions of septic mice. Redox biology.

[22] Li X, Kordsmeier J, Nookaew I, Kim HN, Xiong J (2022). Piezo1 stimulates mitochondrial function via cAMP signaling. FASEB journal: official publication of the Federation of American Societies for Experimental Biology.

[23] Sugimoto A, Iwata K, Kurogoushi R, Tanaka M, Nakashima Y, Yamakawa Y (2023). C-terminus of PIEZO1 governs Ca(2+) influx and intracellular ERK1/2 signaling pathway in mechanotransduction. Biochemical and biophysical research communications.

[24] Yan Z, He Z, Jiang H, Zhang Y, Xu Y, Zhang Y (2023). TRPV4-mediated mitochondrial dysfunction induces pyroptosis and cartilage degradation in osteoarthritis via the Drp1-HK2 axis. International immunopharmacology.

[25] Lin Z, Xu G, Lu X, Wang H, Lu F, Xia X (2025). Piezo1 exacerbates inflammation-induced cartilaginous endplate degeneration by activating mitochondrial fission via the Ca(2+)/CaMKII/Drp1 axis. Aging cell.

[26] Hong W, Xu XY, Qiu ZH, Gao JJ, Wei ZY, Zhen L (2015). Sirt1 is involved in decreased bone formation in aged apolipoprotein E-deficient mice. Acta pharmacologica Sinica.

[27] Yang C, Yang W, He Z, Guo J, Yang X, Wang R (2021). Kaempferol Alleviates Oxidative Stress and Apoptosis Through Mitochondria-dependent Pathway During Lung Ischemia-Reperfusion Injury. Frontiers in pharmacology.

[28] Eaton JS, Lin ZP, Sartorelli AC, Bonawitz ND, Shadel GS (2007). Ataxia-telangiectasia mutated kinase regulates ribonucleotide reductase and mitochondrial homeostasis. The Journal of clinical investigation.

[29] Malik AN, Shahni R, Rodriguez-de-Ledesma A, Laftah A, Cunningham P (2011). Mitochondrial DNA as a non-invasive biomarker: accurate quantification using real time quantitative PCR without co-amplification of pseudogenes and dilution bias. Biochemical and biophysical research communications.

[30] Li Q, Gao Z, Chen Y, Guan MX (2017). The role of mitochondria in osteogenic, adipogenic and chondrogenic differentiation of mesenchymal stem cells. Protein & cell.

[31] Wang L, You X, Zhang L, Zhang C, Zou W (2022). Mechanical regulation of bone remodeling. Bone research.

[32] Coste B, Xiao B, Santos JS, Syeda R, Grandl J, Spencer KS (2012). Piezo proteins are pore-forming subunits of mechanically activated channels. Nature.

[33] Ma S, Dubin AE, Zhang Y, Mousavi SAR, Wang Y, Coombs AM (2021). A role of PIEZO1 in iron metabolism in mice and humans. Cell.

[34] Liu Y, Yang Z, Na J, Chen X, Wang Z, Zheng L (2025). In vitro stretch modulates mitochondrial dynamics and energy metabolism to induce smooth muscle differentiation in mesenchymal stem cells. FASEB journal: official publication of the Federation of American Societies for Experimental Biology.

[35] Zhang D, Lin W, Jiang S, Deng P, Liu L, Wang Q (2023). Lepr-Expressing PDLSCs Contribute to Periodontal Homeostasis and Respond to Mechanical Force by Piezo1. Advanced science (Weinheim, Baden-Wurttemberg, Germany).

[36] Liu F, Yuan L, Li L, Yang J, Liu J, Chen Y (2023). S-sulfhydration of SIRT3 combats BMSC senescence and ameliorates osteoporosis via stabilizing heterochromatic and mitochondrial homeostasis. Pharmacological research.

[37] Nunnari J, Suomalainen A (2012). Mitochondria: in sickness and in health. Cell.

[38] Xu Y, Chang L, Chen Y, Dan Z, Zhou L, Tang J (2024). USP26 Combats Age-Related Declines in Self-Renewal and Multipotent Differentiation of BMSC by Maintaining Mitochondrial Homeostasis. Advanced science (Weinheim, Baden-Wurttemberg, Germany).

[39] Jiang M, Zhang YX, Bu WJ, Li P, Chen JH, Cao M (2023). Piezo1 channel activation stimulates ATP production through enhancing mitochondrial respiration and glycolysis in vascular endothelial cells. British journal of pharmacology.

[40] Bartolák-Suki E, Imsirovic J, Parameswaran H, Wellman TJ, Martinez N, Allen PG (2015). Fluctuation-driven mechanotransduction regulates mitochondrial-network structure and function. Nature materials.

[41] Mousawi F, Peng H, Li J, Ponnambalam S, Roger S, Zhao H (2020). Chemical activation of the Piezo1 channel drives mesenchymal stem cell migration via inducing ATP release and activation of P2 receptor purinergic signaling. Stem cells (Dayton, Ohio).

[42] Li X, Han L, Nookaew I, Mannen E, Silva MJ, Almeida M (2019). Stimulation of Piezo1 by mechanical signals promotes bone anabolism. eLife.

[43] Wang B, Shao W, Zhao Y, Li Z, Wang P, Lv X (2024). Radial extracorporeal shockwave promotes osteogenesis-angiogenesis coupling of bone marrow stromal cells from senile osteoporosis via activating the Piezo1/CaMKII/CREB axis. Bone.

[44] Stapleton D, Mitchelhill KI, Gao G, Widmer J, Michell BJ, Teh T (1996). Mammalian AMP-activated protein kinase subfamily. The Journal of biological chemistry.

[45] Deng M, Yang X, Qin B, Liu T, Zhang H, Guo W (2016). Deubiquitination and Activation of AMPK by USP10. Molecular cell.

[46] Radak Z, Suzuki K, Posa A, Petrovszky Z, Koltai E, Boldogh I (2020). The systemic role of SIRT1 in exercise mediated adaptation. Redox biology.

[47] Wang H, Hu Z, Wu J, Mei Y, Zhang Q, Zhang H (2019). Sirt1 Promotes Osteogenic Differentiation and Increases Alveolar Bone Mass via Bmi1 Activation in Mice. Journal of bone and mineral research: the official journal of the American Society for Bone and Mineral Research.

[48] Sheng SR, Wu YH, Dai ZH, Jin C, He GL, Jin SQ (2023). Safranal inhibits estrogen-deficiency osteoporosis by targeting Sirt1 to interfere with NF-κB acetylation. Phytomedicine: international journal of phytotherapy and phytopharmacology.

[49] Zhu C, Ding H, Shi L, Zhang S, Tong X, Huang M (2023). Exercise improved bone health in aging mice: a role of SIRT1 in regulating autophagy and osteogenic differentiation of BMSCs. Frontiers in endocrinology.

[50] Chen H, Fan W, He H, Huang F (2022). PGC-1: a key regulator in bone homeostasis. Journal of bone and mineral metabolism.

[51] Ono T, Denda R, Tsukahara Y, Nakamura T, Okamoto K, Takayanagi H (2022). Simultaneous augmentation of muscle and bone by locomomimetism through calcium-PGC-1α signaling. Bone research.

[52] Yu B, Huo L, Liu Y, Deng P, Szymanski J, Li J (2018). PGC-1α Controls Skeletal Stem Cell Fate and Bone-Fat Balance in Osteoporosis and Skeletal Aging by Inducing TAZ. Cell stem cell.

[53] Pang J, Yin L, Jiang W, Wang H, Cheng Q, Jiang Z (2024). Sirt1-mediated deacetylation of PGC-1α alleviated hepatic steatosis in type 2 diabetes mellitus via improving mitochondrial fatty acid oxidation. Cellular signalling.

[54] Gerhart-Hines Z, Rodgers JT, Bare O, Lerin C, Kim SH, Mostoslavsky R (2007). Metabolic control of muscle mitochondrial function and fatty acid oxidation through SIRT1/PGC-1alpha. The EMBO journal.

[55] Li L, Mühlfeld C, Niemann B, Pan R, Li R, Hilfiker-Kleiner D (2011). Mitochondrial biogenesis and PGC-1α deacetylation by chronic treadmill exercise: differential response in cardiac and skeletal muscle. Basic research in cardiology.

[56] Liu L, Li Y, Wang J, Zhang D, Wu H, Li W (2021). Mitophagy receptor FUNDC1 is regulated by PGC-1α/NRF1 to fine tune mitochondrial homeostasis. EMBO reports.

[57] Li X, Wang X, Zhang C, Wang J, Wang S, Hu L (2022). Dysfunction of metabolic activity of bone marrow mesenchymal stem cells in aged mice. Cell proliferation.

