# Supplementary material for: Piezo1-driven mechanotransduction regulates mitochondrial biogenesis by AMPK/SIRT1-mediated PGC-1α deacetylation to ameliorate bone loss in disuse osteoporosis
Source: Int J Biol Sci. 2026 Jan 1;22(1):308–26. doi: 10.7150/ijbs.124043 (PMC12681831; doi:10.7150/ijbs.124043)
Supplement: Supplementary file 1 — Supplementary figures and tables. [file ijbsv22p0308s1.pdf]

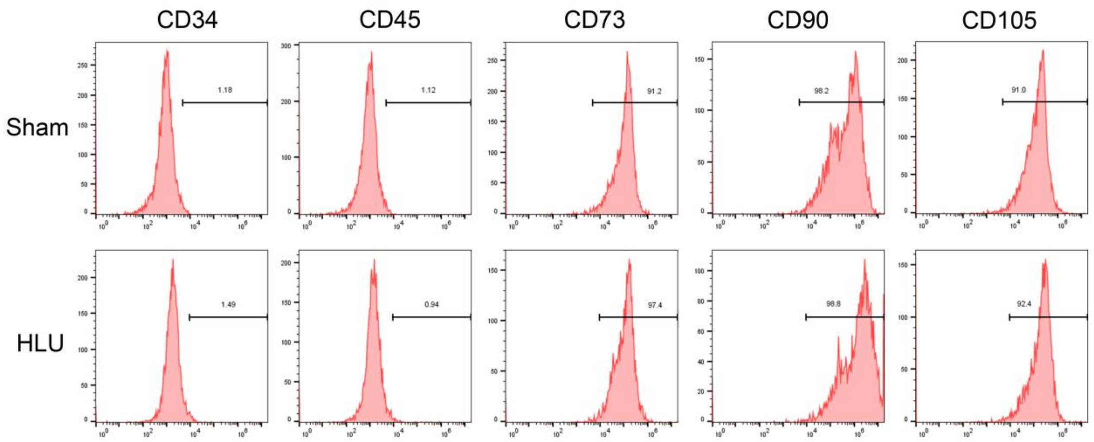

Figure S1. The BMSCs identification results of flow cytometry.

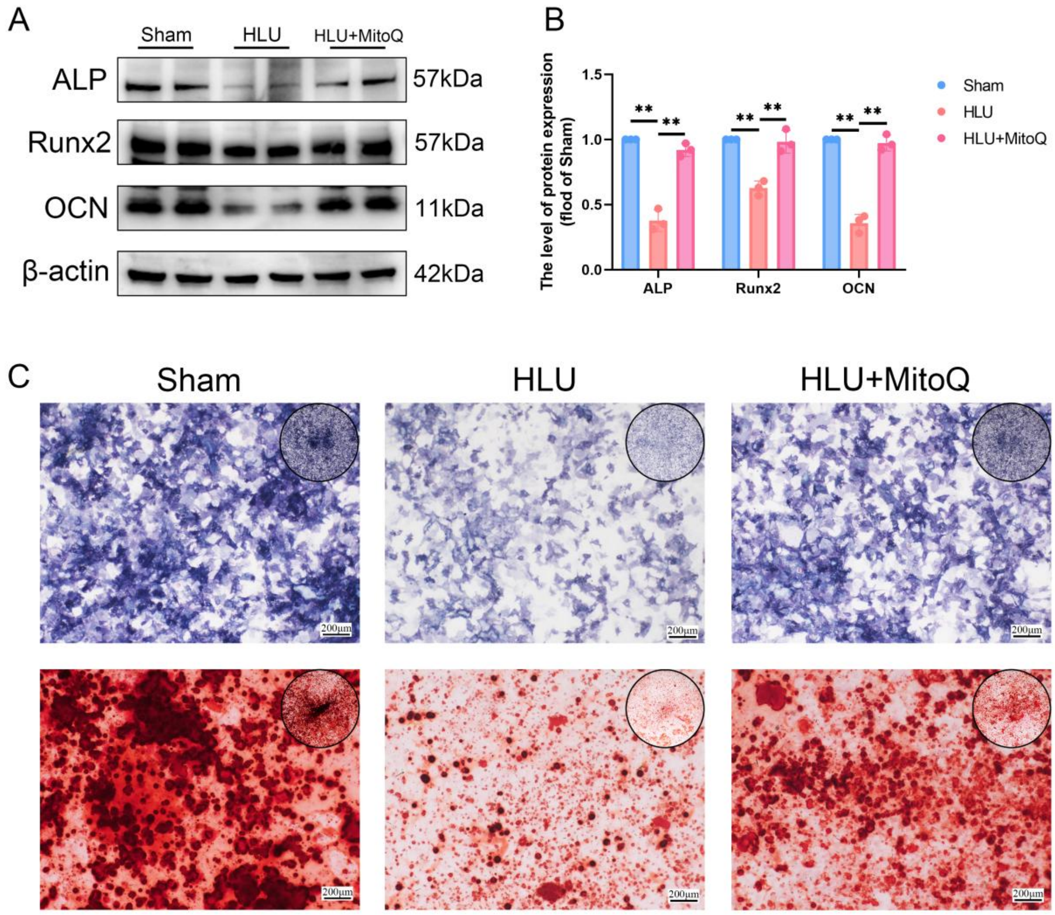

Figure S2. MitoQ restores the osteogenic differentiation impaired by mechanical unloading. (A-B) Western blot analysis of ALP, Runx2, and Ocn protein expression levels. Mean ± SD, n = 3. $*p < 0.05$, $**p < 0.01$. (C) Representative images alkaline phosphatase and alizarin red staining. Scale bar = 200 μm.

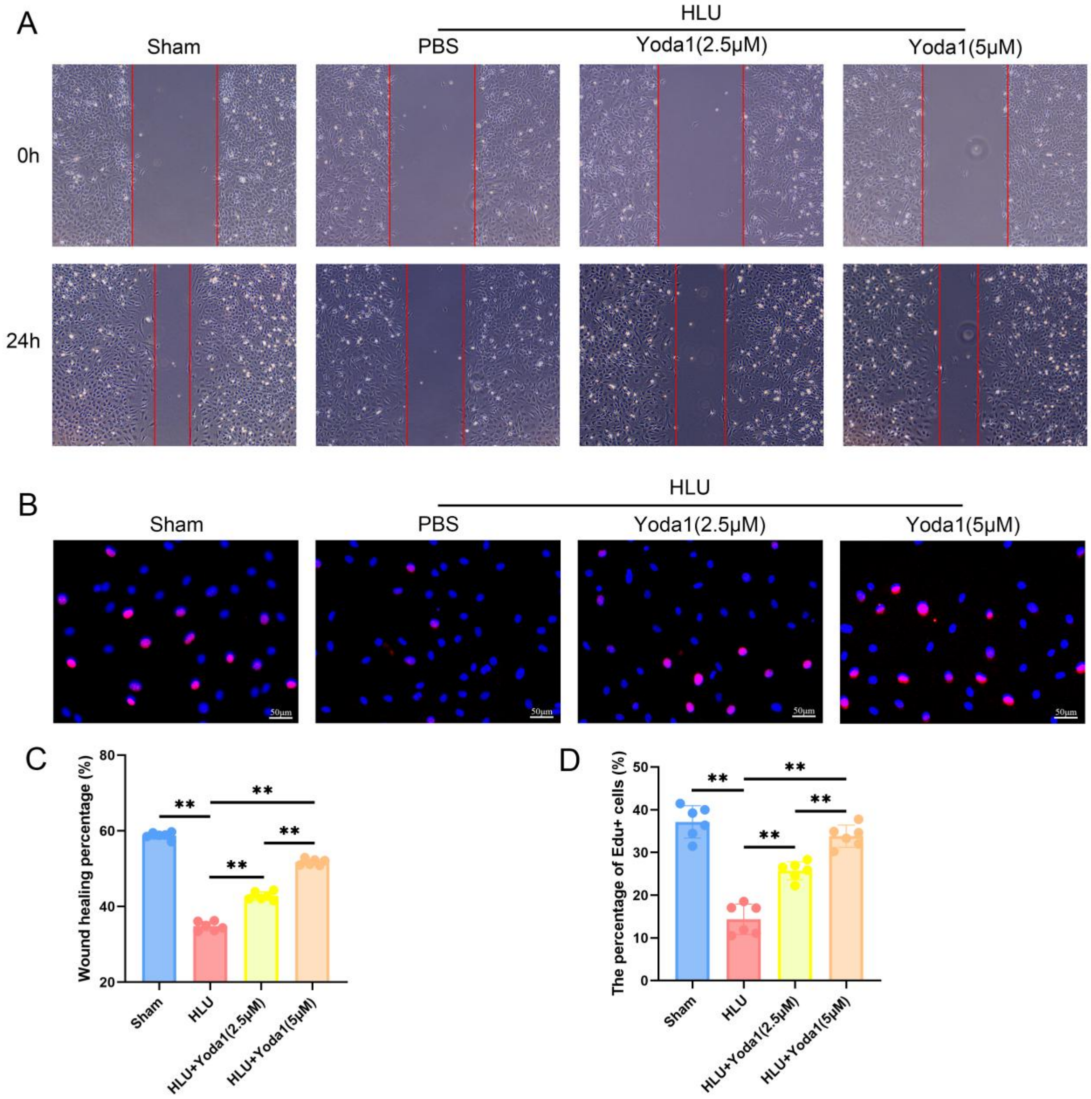

Figure S3. **Yoda1 promotes the proliferation of BMSCs in vitro.** (A, C) Representative images and quantification analysis of scratch experiment. Mean ± SD, n = 6. *p < 0.05, **p < 0.01. (B, D) Representative immunofluorescence images and quantification analysis of Edu-positive cells. Scale bar = 50μm. Mean ± SD, n = 6. *p < 0.05, **p < 0.01.

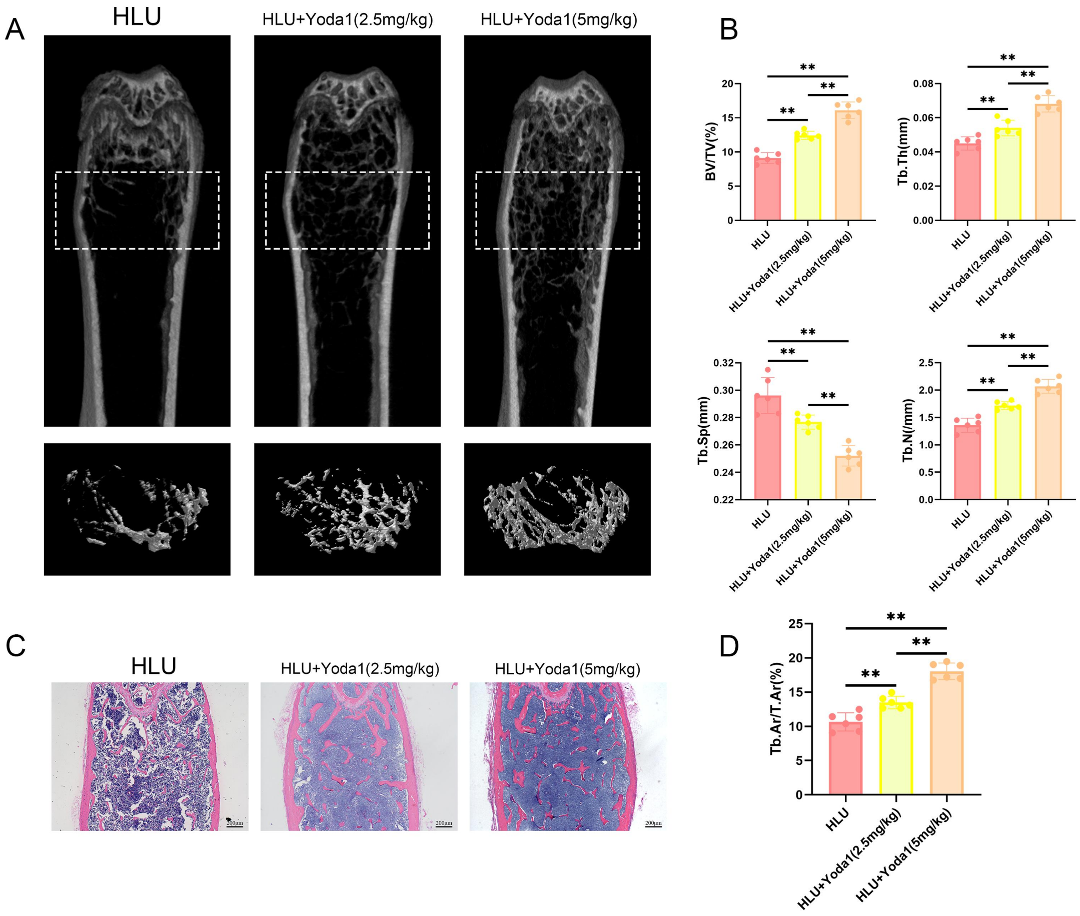

Figure S4. Yoda1 promotes bone formation in vivo. (A) Representative 3D micro-CT reconstructions of distal femoral trabecular architecture. (B) Quantitative analysis of trabecular bone parameters: bone volume fraction (BV/TV), trabecular number (Tb.N), trabecular thickness (Tb.Th), and trabecular separation (Tb.Sp). Data: Mean ± SD, n = 6, *p< 0.05, **p < 0.01. (C) Representative H&E-stained sections of distal femurs. Scale bar = 200 μm. (D) Quantitative analysis of B.Ar/T.Ar. B.Ar = bone area; T.Ar = total area. Data: Mean ± SD, n = 6, *p< 0.05, **p < 0.01.

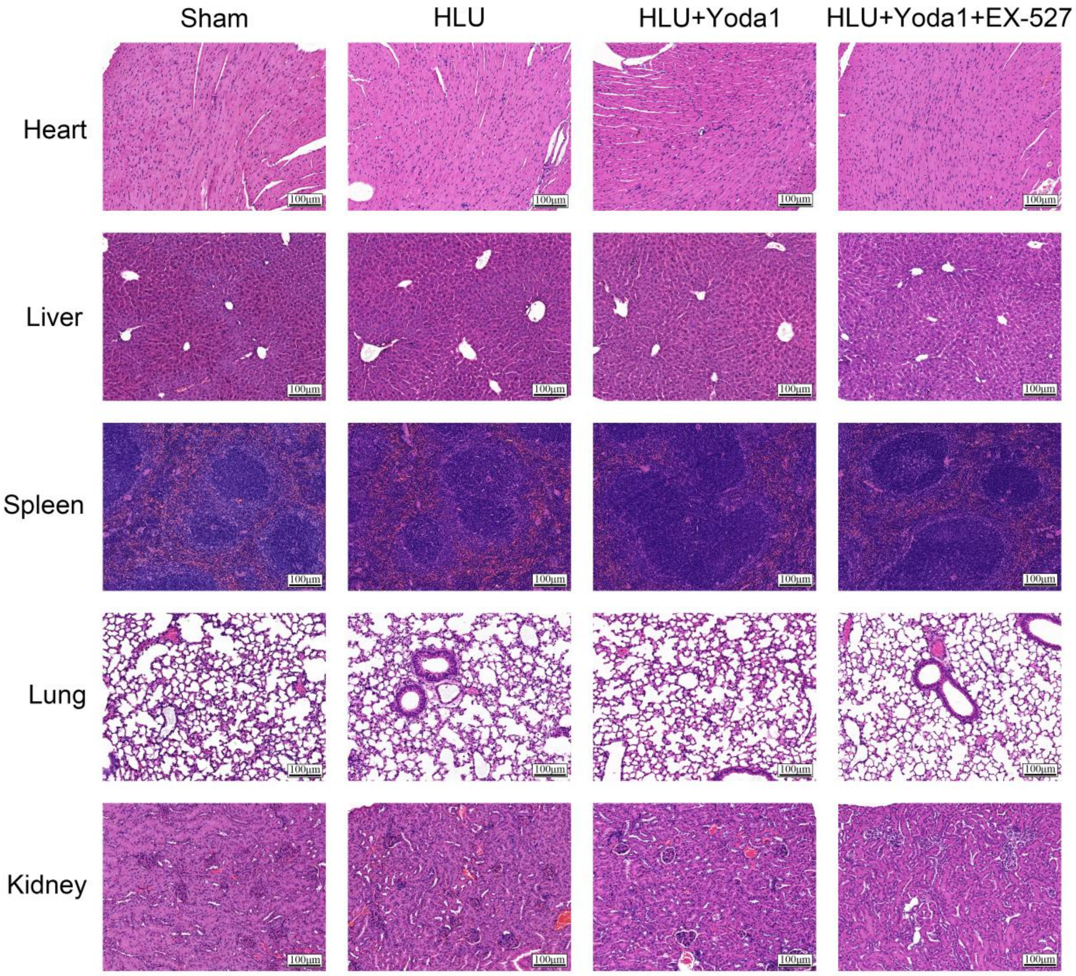

Figure S5. HE staining of heart, liver, spleen, lung, and kidney tissues from Sham, HLU, HLU + Yoda1, and HLU + Yoda1 + EX-527 groups. Scale bar: 100μm.

Table S1. The antibodies.

Antibody	Manufacturer	Application
PGC-1α	Proteintech Group (66369-1-Ig)	WB (1:1000), ICC (1:500)
Piezo1	Proteintech Group (15939-1-AP)	WB (1:200), IHC (1:100)
TFAM	Proteintech Group (22586-1-AP)	WB (1:1000), ICC (1:200), IHC (1:500)
Runx2	Proteintech Group (20700-1-AP)	WB (1:200), ICC (1:100)
Pan-Acetylation	Proteintech Group (66289-1-Ig)	WB (1:500), ICC (1:100)
NRF1	Proteintech Group (66832-1-Ig)	WB (1:1000), ICC (1:200)
Cleaved Caspase 3	Proteintech Group (25128-1-AP)	WB (1:500)
Bax	Proteintech Group (50599-2-Ig)	WB (1:500)
Bcl2	Proteintech Group (26593-1-AP)	WB (1:500)
SIRT1	Proteintech Group (13161-1-AP)	WB (1:1000)
Beta Actin	Proteintech Group (66009-1-Ig)	WB (1:10000)
CaMKII	Cell Signaling Technology (11945)	WB (1:500)
P-CaMKII	Cell Signaling Technology (12716)	WB (1:500)
AMPK	Immunoway (YT0216)	WB (1:500)
P-AMPK	Immunoway (YM8689)	WB (1:1000)
ALP	Affinity Biosciences (DF6225)	WB (1:500)
TOM20	ABclonal (A19403)	ICC (1:200)
dsDNA	Santa Cruz (sc-58749)	ICC (1:200)
osteocalcin	Santa Cruz (sc-365797)	WB (1:500), IF (1:200)
SIRT1	Servicebio (GB11171)	IF/ICC (1:200)
HRP-conjugated Goat Anti-Mouse IgG(H+L)	Proteintech Group (SA00001-1)	WB (1:5000)
HRP-conjugated Goat Anti-Rabbit IgG(H+L)	Proteintech Group (SA00001-2)	WB (1:5000)
Goat anti-Rabbit IgG (H+L) Cross-Adsorbed Secondary Antibody, Alexa Fluor™ 488	Invitrogen (A-11008)	IF/ICC (1:200)
Goat anti-Mouse IgG (H+L) Cross-Adsorbed Secondary Antibody, Alexa Fluor™ 594	Invitrogen (A-11005)	IF/ICC (1:200)
HRP-conjugated goat anti-rabbit/mouse IgG secondary antibody	Servicebio (G1303)	IHC (immediately)

WB: Western Blot, IHC: Immunohistochemistry, IF: Immunofluorescence, ICC: Immunocytochemistry

Table S2. Primers used in mtDNA copy number assay.

Gene	Sequence 5'-3'	Species
COX2	ATAACCGAGTCGTTCTGCCAAT	Mouse
	TTTCAGAGCATTGGCCATAGAA	
RPS18	TGTGTTAGGGGACTGGTGGACA	Mouse
	CATCACCCACTTACCCCCAAAA	
